# Tryptophan residues in TDP-43 and SOD1 modulate the cross-seeding and toxicity of SOD1

**DOI:** 10.1016/j.jbc.2024.107207

**Published:** 2024-03-22

**Authors:** Edward Pokrishevsky, Michéle G. DuVal, Luke McAlary, Sarah Louadi, Silvia Pozzi, Andrei Roman, Steven S. Plotkin, Anke Dijkstra, Jean-Pierre Julien, W. Ted Allison, Neil R. Cashman

**Affiliations:** 1 Department of Medicine, Djavad Mowafaghian Centre for Brain Health, University of British Columbia, Vancouver, British Columbia, Canada; 2 Department of Biological Sciences, Centre for Prions & Protein Folding Disease, University of Alberta, Edmonton, Alberta, Canada; 3 Department of Physics and Astronomy, University of British Columbia, Vancouver, British Columbia, Canada; 4 Department of Psychiatry and Neuroscience, University of Laval, Québec, Quebec, Canada; 5 CERVO Brain Research Center, Québec, Quebec, Canada; 6 Department of Pathology, Amsterdam Neuroscience, Amsterdam University Medical Centre, Amsterdam, The Netherlands

**Keywords:** ALS, TDP-43, SOD1, protein aggregation, prion-like propagation, cross-seeding

## Abstract

Amyotrophic lateral sclerosis (ALS) is a fatal neurodegenerative disease of motor neurons. Neuronal superoxide dismutase-1 (SOD1) inclusion bodies are characteristic of familial ALS with SOD1 mutations, while a hallmark of sporadic ALS is inclusions containing aggregated WT TAR DNA-binding protein 43 (TDP-43). We show here that co-expression of mutant or WT TDP-43 with SOD1 leads to misfolding of endogenous SOD1 and aggregation of SOD1 reporter protein SOD1^G85R^-GFP in human cell cultures and promotes synergistic axonopathy in zebrafish. Intriguingly, this pathological interaction is modulated by natively solvent-exposed tryptophans in SOD1 (tryptophan-32) and TDP-43 RNA-recognition motif RRM1 (tryptophan-172), in concert with natively sequestered TDP-43 N-terminal domain tryptophan-68. TDP-43 RRM1 intrabodies reduce WT SOD1 misfolding in human cell cultures, *via* blocking tryptophan-172. Tryptophan-68 becomes antibody-accessible in aggregated TDP-43 in sporadic ALS motor neurons and cell culture. 5-fluorouridine inhibits TDP-43–induced G85R-GFP SOD1 aggregation in human cell cultures and ameliorates axonopathy in zebrafish, *via* its interaction with SOD1 tryptophan-32. Collectively, our results establish a novel and potentially druggable tryptophan-mediated mechanism whereby two principal ALS disease effector proteins might directly interact in disease.

Amyotrophic lateral sclerosis (ALS) is characterized by progressive paralysis of the muscles of the limbs, speech, swallowing, and respiration, usually leading to death within 2 to 5 years. Although the familial forms of the disease can be caused by mutation of ∼20 genes, including Cu/Zn superoxide dismutase (*SOD1*) (1) and *trans*-activation response DNA-binding protein (*TARDBP*, encoding the protein TDP-43) (2), pathologically aggregated WT TDP-43 inclusions are a hallmark of all non-SOD1/fused in sarcoma–associated ALS (3, 4). While mutations in SOD1 lead to robust intracellular aggregates of the protein in patient neurons, misfolded WT SOD1 can be detected by various SOD1 misfolding-specific antibodies in some sporadic ALS cases, in a granular and homogeneously distributed pattern (5, 6, 7, 8, 9). We have previously shown that transfection-mediated overexpression of mutant and WT TDP-43 induce the misfolding of endogenous human WT SOD1 in cultured cell lines, as well as in primary spinal cord cells derived from human WT SOD1 transgenic mice (5). The mechanisms whereby TDP-43 can induce misfolding of WT SOD1 has been unknown.

We previously reported that the prion-like seeding and propagation of human WT SOD1 misfolding is modulated by a tryptophan–tryptophan interaction in neighboring SOD1 molecules mediated by the single monomer tryptophan at position 32 in SOD1 (Trp32) (10, 11, 12). Also, Trp32 modulates mutant SOD1 aggregation in cultured cells, as well as zebrafish axonopathy *in vivo* (13, 14). Furthermore, drugs that interact with Trp32 (15, 16) can block propagated SOD1 aggregation in cell cultures and its toxicity *in vivo* (13, 14). Trp32 in SOD1 is estimated to be more natively solvent exposed than 90% of other tryptophans in the human structural proteome (10), an oddity consistent with pathological functionality, such as mediating intermolecular interactions between SOD1 and other molecules. Indeed, an aberrant interaction between, and co-aggregation of, mutant SOD1 with the stress-granule modulating protein G3BP1 depends on a three-way collaboration of Trp32 in SOD1 and a pair of aromatic residues (Phe380 and Phe382) in G3BP1 (17). Given the potential role of solvent-exposed Trp in the misfolding and aggregation of SOD1, we speculated here that cross-seeding of human SOD1 by TDP-43 might be dependent on early interactions along the misfolding pathway between solvent-exposed Trp residues in both proteins. TDP-43 possesses six Trp residues, five of which are substantially solvent-exposed in the native structure: two in the structured RNA recognition motif (RRM1) responsible for binding DNA and RNA (PDB: 4IUF (18), 4Y0F (19)) and three in the poorly structured low complexity C-terminal domain. The sixth tryptophan, Trp68, resides in the N-terminal domain (NTD) and is not solvent accessible in its natively folded structure (20). We now show that the NTD Trp68 becomes antibody-accessible when aggregated in cultured cells and in sporadic ALS post-mortem spinal cord. We also demonstrate that TDP-43–induced SOD1 misfolding, aggregation, and toxicity *in vivo* and *in vitro* are modulated by a three-way collaboration between two natively exposed tryptophans (SOD1 Trp32 and TDP-43 RRM1 Trp172) and the misfolding-exposed TDP-43 Trp68. These findings are further supported by the blockade of WT SOD1 misfolding by intrabodies directed at TDP-43 RRM1 and by inhibition of cellular aggregation and zebrafish axonopathy by the Trp-interacting small molecule 5-fluorouridine.

## Results

### Interaction between tryptophan residues mediates the intermolecular cross-seeding of SOD1 by TDP-43 in cultured cells

**Figure 1:**
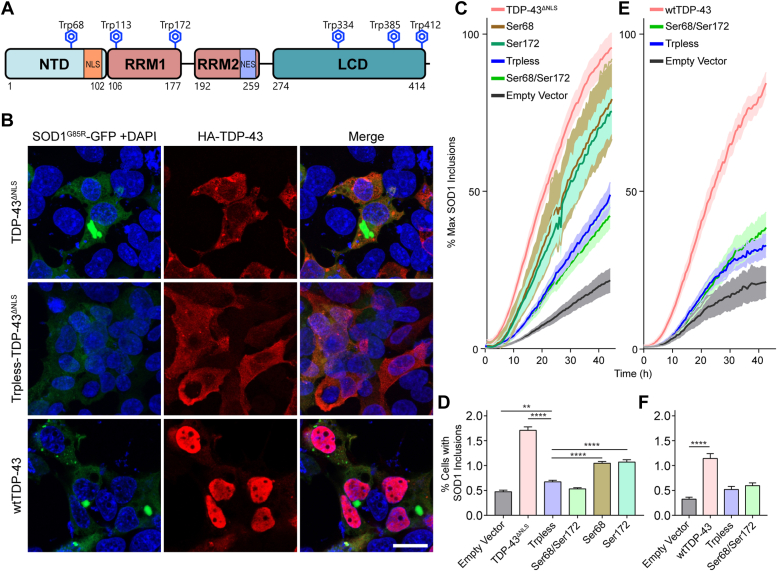
**Tryptophan residues in wtTDP-43 and TDP-43**^Δ**NLS**^**modulate the aggregation of mutant SOD1**^**G85R**^**-GFP in cultured cells.***A*, schematic representation of the TDP-43 structural domains and location of its six Trp residues. *B*, TDP-43 variants (*red*) cross-seed aggregation of SOD^G85R^-GFP reporter protein (*green*), viewed 48 h after co-transfection into HEK293FT cells. TDP-43^ΔNLS^ with all Trp residues mutated to serine (Trpless-TDP-43^ΔNLS^) is abundantly expressed but does not induce SOD1^G85R^-GFP aggregation. Scale bar represents 20 μm. *C*, time-course analysis of induced SOD1^G85R^-GFP aggregate abundance in HEK293FT cells from 24 to 72 h post co-transfection with TDP-43^ΔNLS^, wherein combinations of Trp residues are mutated to serine (Ser). Trp68 and Trp172 in TDP-43 modulate the induction of SOD1^G85R^-GFP aggregation. *E*, as per transfections of panel C, except using WT TDP-43 plasmid variants. *D* and *F*, percent of cells with detectable SOD1^G85R^-GFP aggregates quantified by flow cytometry shows more cross seeding of SOD1^G85R^-GFP by TDP-43^ΔNLS^ (*D*) or WT TDP-43 (*F*) constructs when compared to their variants bearing Trp to Ser mutation(s). Statistical significance was determined using one-way ANOVA and Dunnett’s test for multiple comparisons (∗∗*p* < 0.01; ∗∗∗∗*p* < 0.0001). Error bars represent SEM; between, 8 and 34 biological replicates were performed for each construct. LCD, low-complexity domain; NES, nuclear export signal; NLS, nuclear localization signal; NTD, N-terminal domain; RRM, RNA recognition motif; RRM, RNA recognition motif; TDP, TAR DNA-binding protein.

We have previously reported that HEK293FT cells overexpressing either WT or mutant nuclear localization signal (ΔNLS) TDP-43 (TDP-43^ΔNLS^) can convert endogenous WT human SOD1 to a misfolded, propagating, and toxic form (5, 13). Since SOD1 seeding and misfolding propagation has been shown to be modulated by its single tryptophan residue in each monomer, Trp32 (10, 13, 14), we speculated that TDP-43 might induce SOD1 misfolding and aggregation *via* one or more of its six Trp residues (Fig. 1*A*). We started exploring this potential mechanism by employing a natively unfolded mutant SOD1^G85R^ that has been shown to be an excellent substrate for misfolding SOD1 seeds *in vitro* and *in vivo* (12, 21, 22). Experimentally, we cotransfected HEK293FT cells with SOD1^G85R^-GFP reporter (13, 22) and WT TDP-43 or nuclear localization signal mutant TDP-43^ΔNLS^ and showed induction of SOD1^G85R^-GFP aggregation by these cytosolically mislocalizing and aggregating TDP-43 variants (Fig. 1*B*), supported by live-cell kinetic analysis (Fig. 1, *C* and *E*). The induction of SOD1^G85R^-GFP aggregation by WT TDP-43 or TDP-43^ΔNLS^ was confirmed independently by flow cytometry analysis showing a significant 3-fold increase in the induction of SOD1^G85R^-GFP aggregation (Fig. 1, *D* and *F*). We next substituted all six of the Trp residues in WT TDP-43 or TDP-43^ΔNLS^ with serine residues (tryptophan-less or “Trpless”). We found that co-expression of Trpless TDP-43^ΔNLS^ or Trpless WT TDP-43, with the reporter protein, SOD1^G85R^-GFP, resulted in a significant decrease of induced SOD1^G85R^-GFP aggregates forming across time as captured by time-lapse live-cell microscopy (Fig. 1, *C* and *E*). This decrease in the seeding of SOD1^G85R^-GFP was confirmed using flow cytometry (Fig. 1, *D* and *F*). Importantly, the Trpless TDP-43 variants were expressed at comparable abundance to TDP-43^ΔNLS^ (Fig. 1*B*).

We next performed combinatorial step-wise Trp to Ser mutagenesis in order to identify which of the six Trp residues in TDP-43 are required for inducing SOD1^G85R^-GFP aggregation (Fig. S1). We identified Trp68 and Trp172 in TDP-43 as modulators for the induction of SOD1^G85R^-GFP aggregation, with their combined mutation to Ser leading to a significant 2-fold decrease in the SOD1^G85R^-GFP aggregation rate (Figs. 1, *C* and *D*, and S2*A*) and a 3-fold reduction in SOD1^G85R^-GFP aggregation measured *via* microscopy and flow cytometry (Fig. 1, *E* and *F*) respectively. Indeed, mutagenesis of both Trp68 and Trp172 reduced SOD1^G85R^-GFP aggregation to levels comparable to Trpless TDP-43^ΔNLS^ or to the vector-only negative control (reporter only; no TDP-43^ΔNLS^ in the co-transfection) (Fig. 1, *C* and *D*). Further Trp substitutions other than Trp68 and Trp172 did not significantly prevent SOD1^G85R^-GFP aggregation, including mutation of Trp113 to produce a triple substitution of the Trp68 and the two RRM1 Trps. Trp to Ser mutations of the three C-terminal Trps in the TDP-43 low-complexity domain, which have been previously shown to participate in TDP-43 self-aggregation (23), did not affect SOD1^G85R^-GFP aggregation in co-transfected HEK293FT cells (Fig. S1). In sum, WT TDP-43 and TDP-43^ΔNLS^ variants can induce SOD1^G85R^-GFP aggregation in HEK293FT cells, by a mechanism that requires two collaborating N-terminal Trp residues Trp68 and Trp172.

### SOD1 and TDP-43 synergize to cause zebrafish motor axonopathy *via* tryptophan residues

**Figure 2:**
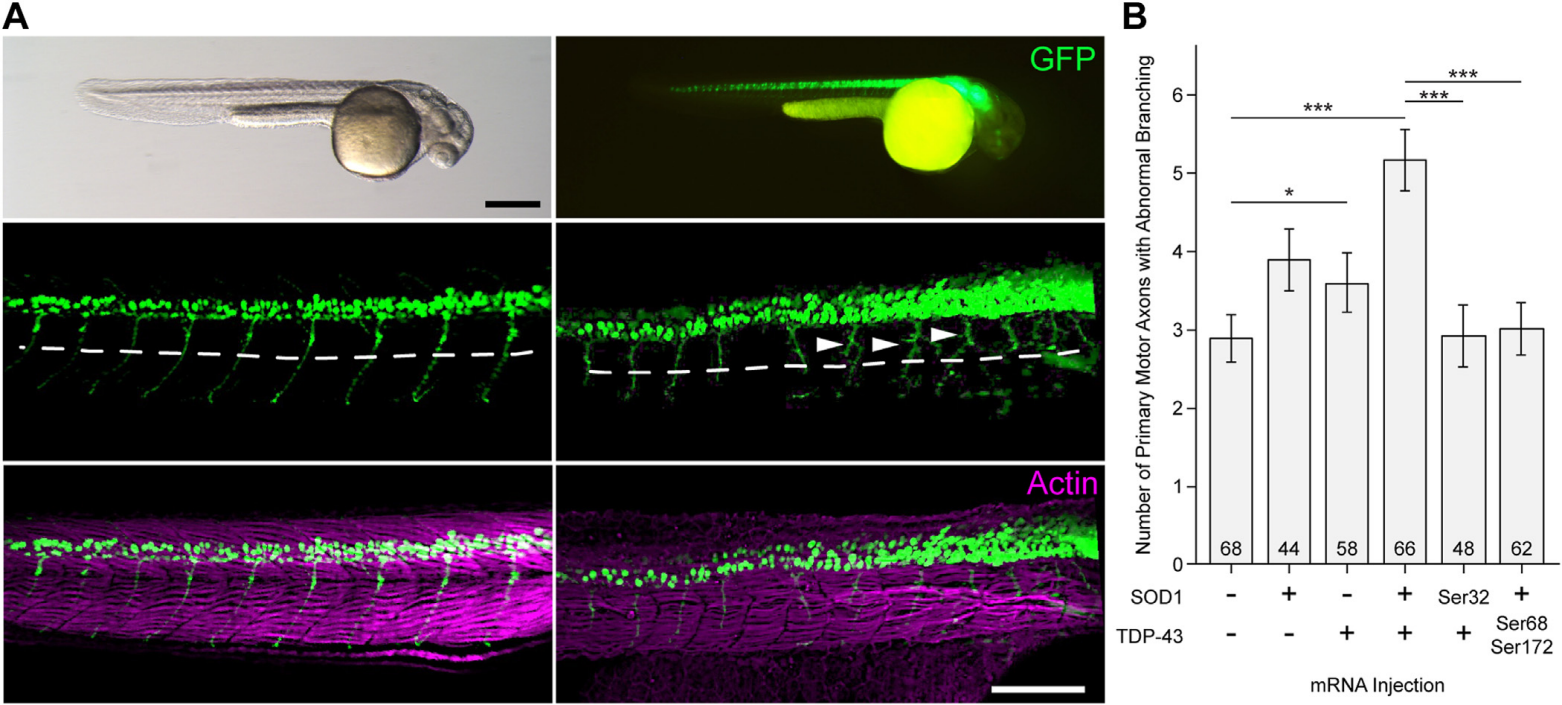
**Tryptophan residues in human SOD1 and TDP-43 proteins mediate their synergistic impact on motor neuron pathology *in vivo*.***A*, *mnx1:GFP* zebrafish (36 hpf, hours post-fertilization) expressing GFP in the motor neurons. Abnormal primary axons (arrowheads) with branches above the ventral notochord (*dashed line*). Muscle actin counterstained in *magenta*. Scale bars represent 0.5 mm (*top left* and *top right*); 200 μm (*bottom* four panels). *B*, human WT SOD1 and WT TDP-43 synergistically induce primary motor neuron axonopathy. Ser68 and Ser172 TDP-43 mutations reduced SOD1-TDP-43–induced axonopathy (*p* = 3.57 × 10^−5^). The SOD1 Trp32Ser mutation also abrogated axonopathy (*p* = 4.54 × 10^−5^) and in combination with Trpless-TDP-43 (*p* = 8.94 × 10^−5^). Error bars indicate SEM. ∗*p* < 0.05, ∗∗*p* < 0.01, ∗∗∗*p* < 0.001 (Kruskall–Wallis test with Mann–Whitney pairwise comparisons): sample sizes are noted at the base of each bar. Mnx1, motor neuron and pancreas homeobox protein 1; SOD1, superoxide dismutase; TDP, TAR DNA-binding protein.

We next sought to explore the possibility that Trp–Trp interactions might modulate TDP-43 and SOD1 folding and toxicity in an *in vivo* model. Despite being an acute model, zebrafish axonopathy has proven to be an informative and tractable proxy for *in vivo* events associated with ALS etiology (24). WT TDP-43 and SOD1 could participate in sporadic ALS, so we assessed zebrafish primary motor axons for abnormal morphology in larvae injected with mRNA encoding human WT SOD1 Trp variants and/or human WT TDP-43 Trp variants. Overexpression of WT human SOD1 or TDP-43 in zebrafish has been previously found to engender abnormal axon branching (14, 24, 25, 26, 27, 28). Assessing axonopathy of primary motor neurons in the zebrafish ALS model allowed us to efficiently compare several Trp to Ser variants of SOD1 and TDP-43 *in vivo*, powered by robust sample sizes. Healthy primary motor axons extend from the spinal cord, past the notochord to innervate the trunk muscles (Fig. 2*A*); these axons rarely exhibit branching above the ventral notochord boundary at 34 to 36 h post fertilization, and abnormal axon morphology includes branching above this boundary (Fig. 2*A*). Abnormal branching may also be associated with muscle fiber patterning (Fig. 2*A*). Overexpression of human WT SOD1 significantly elevated axonopathy by 1.3 fold over controls, similarly to previous reports (14, 27, 28), and expression of human WT TDP-43 in isolation increased axonopathy by 15% (Fig. 2*B*). Compared to mRNA controls, the co-expression of SOD1 and TDP-43 significantly increased axonopathy by approximately 1.8 fold, the synergy of which is consistent with our hypothesis that TDP-43 induces SOD1 misfolding and toxicity (Fig. 2*B*).

Guided by the results from the HEK293FT cell system detailed above, we next proceeded with substituting Trp residues in WT SOD1 and/or W TDP-43 in combination to determine the impact of the Trps on zebrafish axonopathy. Substitution of Trp32 in SOD1 (SOD1^Trp32Ser^) abolished SOD1-TDP-43–induced axonopathy (*p* = 8.94 × 10^−5^) (Fig. 2*B*), consistent with cell-based findings demonstrating that Trp32 is necessary for SOD1 to be induced to cross-seed by TDP-43. Again, we performed step-wise Trp substitutions in TDP-43 and found these to lead to statistically significant cumulative reductions in axonopathy, back to control mRNA levels. Mutation of Trp68 and Trp172 showed complete abrogation of the SOD1-TDP-43 synergistic impact (*p* = 3.57 × 10^−5^) (Fig. 2*B*). These *in vivo* axonopathy results are consistent with the effect of Trp substitutions on SOD1 misfolding/aggregation as assessed in HEK293FT cells cotransfected with SOD1^G85R^-GFP and TDP-43 variants (Figs. 1 and S2).

### Tryptophan 68 in TDP-43 NTD is exposed during misfolding/aggregation

**Figure 3:**
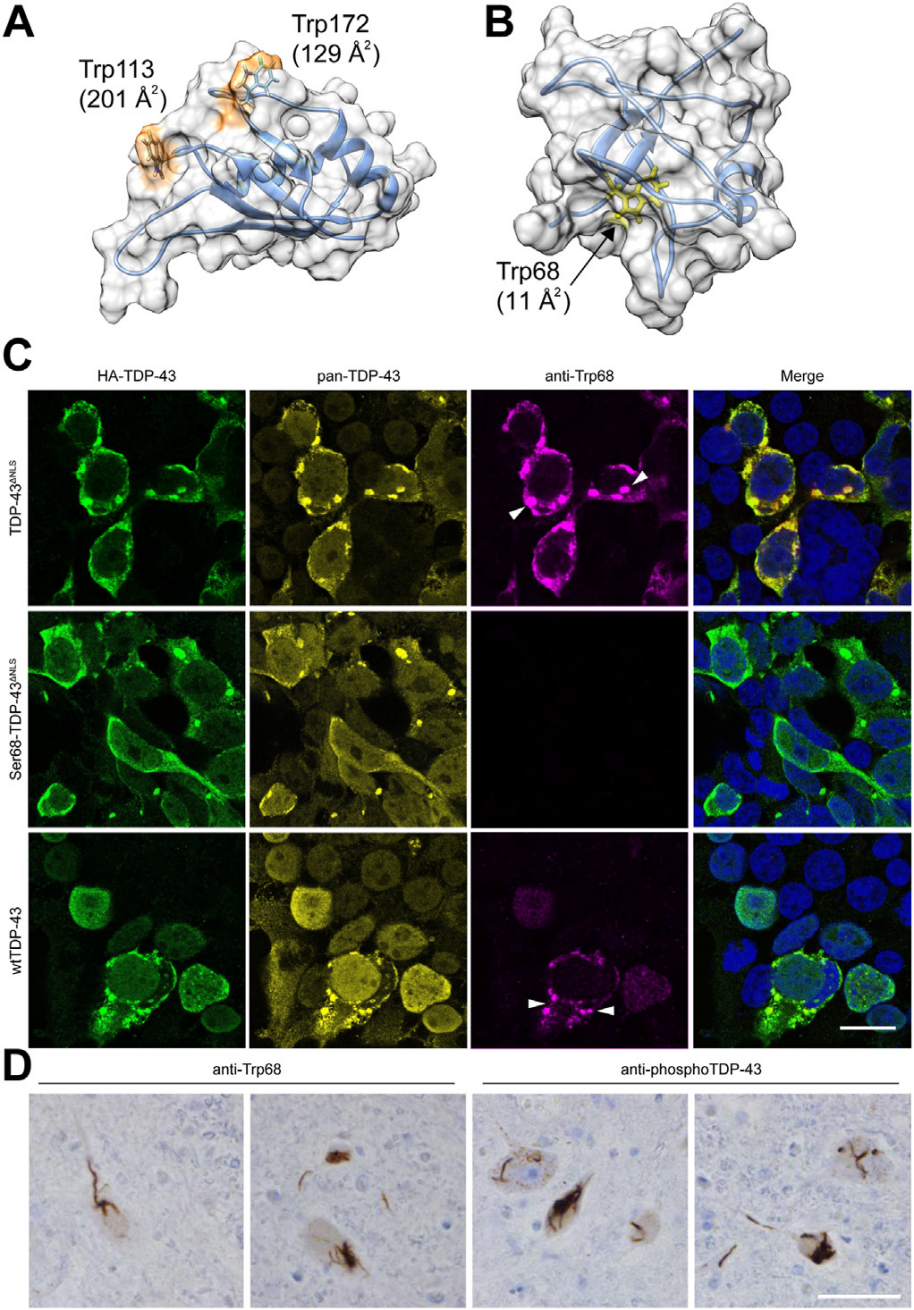
**Trp68 is exposed in cytoplasmic TDP-43 aggregates in cultured cells and in sporadic ALS motor neurons.***A*, representative centroid structure of the RRM1 domain of TDP-43, obtained from equilibrium molecular dynamics (MD) simulation. Trps 113 and 172 are significantly exposed compared to most native structures in the protein databank (11). *B*, representative centroid structure of the N-terminal domain of TDP-43, obtained from equilibrium molecular dynamics simulation. Trp68 is nearly fully buried: only about 9% of its tripeptide Gly-Trp-Gly value of 247 Angstrom^2^ is exposed in the centroid structure. *C*, the affinity-purified rabbit polyclonal anti-Trp68 antibody (*red*) was tested for reactivity and specificity in cells transfected with different TDP-43 constructs, then fixed and stained 48 h later. A mouse pan-TDP-43 antibody against the C-terminal domain and a chicken anti-HA-tag antibody were used to test co-localization with TDP-43 (*yellow*) and HA-TDP-43 (*green*), respectively. The anti-Trp68 antibody specifically recognizes mislocalized cytoplasmic TDP-43 aggregates in TDP-43^ΔNLS^-transfected cells but not those lacking Trp68 (Ser68 TDP-43^ΔNLS^) nor does it recognize nuclear TDP-43. The composite images are a merge between nuclear, anti-Trp68, and anti-HA-tag staining. Scale bar represents 20 μm. *D*, representative images of TDP-43 pathology in ALS cervical spinal cord sections from two subjects immunostained with anti-Trp68 in paraffin-embedded tissue. The *left panel* shows the anti-Trp68 antibody and *right* the commercial pTDP-43 antibody. Motor neurons show thread-like inclusions using the anti-Trp68 antibody and the commercial pTDP-43 antibody, but no immunoreactivity is observed of natively folded TDP-43 in neuronal or glial nuclei Scale bar represents 50 μm. ALS, amyotrophic lateral sclerosis; RRM, RNA recognition motif; TDP, TAR DNA-binding protein.

**Figure 4:**
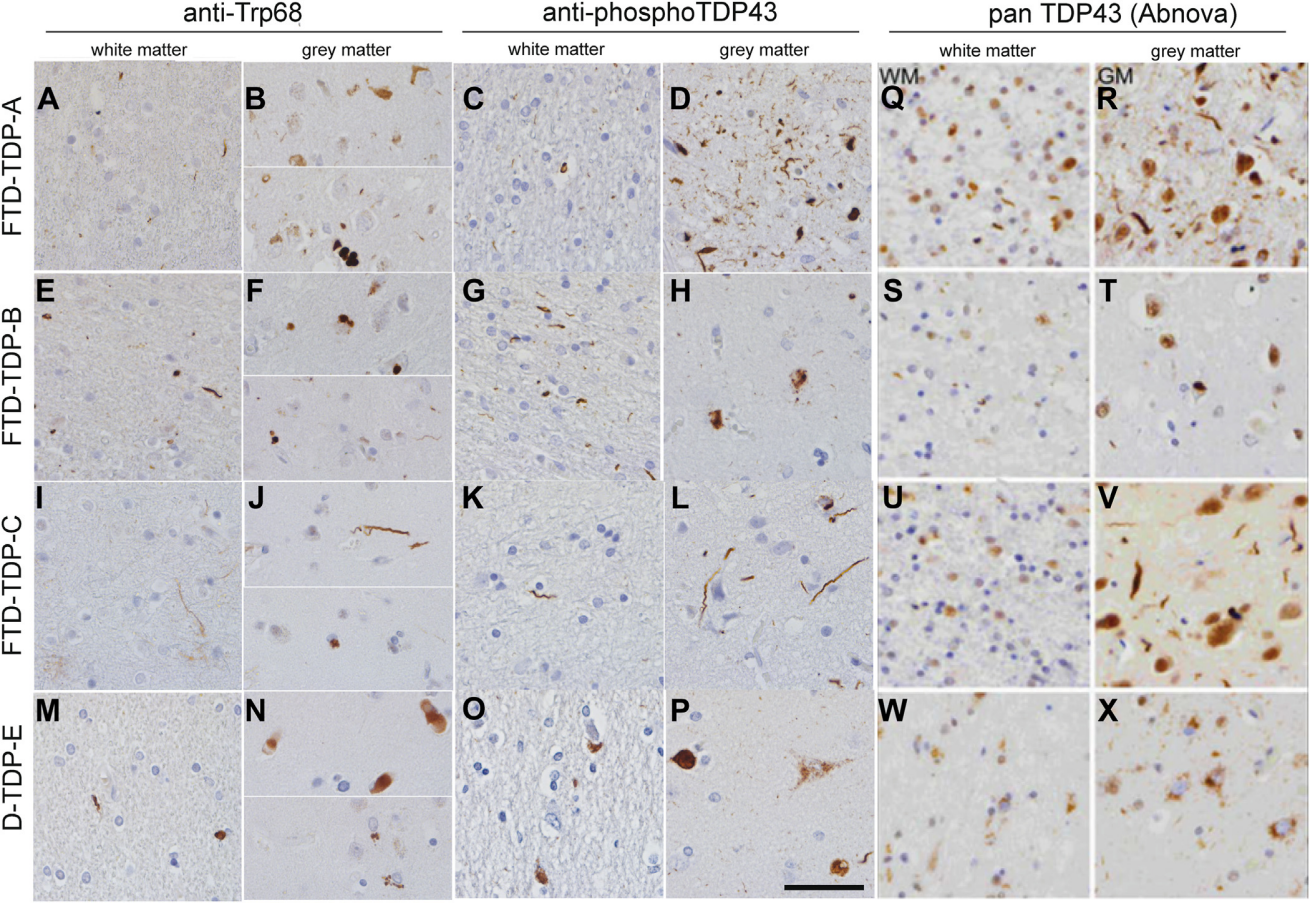
**Trp68 is exposed in TDP-43 aggregates in *gray* and *white* matter in TDP43 subtypes of FTD.** TDP-43 pathology in sections from the anterior cingulate cortex of FTD-TDP subtypes *A*, *B*, *C*, and *E* immunostained with anti-Trp68 (*left* column), commercial phospho-TDP-43 (*central* column), and commercial pan-TDP-43 (*right* column) in paraffin-embedded tissue. FTD-TDP-A shows thread-like inclusions in the *white* matter using the anti-Trp68 antibody (*A*) and the commercial pTDP-43 antibody (*C*). In the *gray* matter, cytoplasmic inclusions are predominantly stained using the anti-Trp68 antibody (*B*) and the pTDP-43 antibody (*D*) that shows abundant staining of cytoplasmic and extracellular aggregates. In FTD-TDP-B, mainly cytoplasmic inclusions are observed in the *white* and *gray* matter using the anti-Trp68 antibody (*E* and *F*) and the commercial pTDP-43 antibody (*G* and *H*). FTD-TDP-C is characterized by long threads in the *white* and *gray* matter. These long threads are seen using the anti-Trp68 antibody (*I* and *J*) and the pTDP-43 antibody (*K* and *L*). FTD-TDP-E is characterized by cytoplasmic and abundant granular TDP-43 aggregation. Using the anti-Trp68 antibody, cytoplasmic and thread-like inclusions are seen in *white* and *gray* matter (*M* and *N*), but no granular staining is observed. The pTDP-43 antibody shows cytoplasmic and granular staining in the *white* and *gray* matter (*O* and *P*). The right column (*Q*–*X*) shows pan-TDP-43 distribution, which does not well correspond to pathological antibodies. Scale bar represents 50 μm. FTD, frontotemporal domain; TDP, TAR DNA-binding protein.

TDP-43 RRM1 Trp172 appears to be solvent accessible on visual inspection of protein database (PDB) RRM1 example structures (Figure 3, Figure 4*A*) 4IUF (18) and 4Y0F (19), and we have calculated that Trp172 solvent accessible surface area is at the 91.8^th^ percentile compared to all Trps within a non-redundant PDB database of 27,015 proteins (29). This number is likely to be even somewhat higher as this dataset includes integral membrane proteins wherein membrane is not included in the calculation. However, TDP-43 Trp68 does not seem to be available for interaction at the TDP-43 molecular surface and appears buried in the native structural core of the NTD (Fig. 3*B*), at the 26.6^th^ percentile for solvent accessible surface area (29). We hypothesized that Trp68 acquires solvent exposure in misfolded/aggregated pathological TDP-43 in order to participate in the cross-seeding of WT SOD1. To test this hypothesis, we developed an affinity-purified rabbit polyclonal immunoglobulin G generated against a linear peptide encompassing Trp68 in the context of its local amino acid sequence (_65_DAGWGNL_71_). Recombinantly expressed TDP-43 NTD demonstrated immunoreactivity with this antibody (named anti-Trp68) in a denaturing immunoblot system, but not in native gel immunoblots (Fig. S3, *A* and *B*), demonstrating that loss of structure of NTD is required for antibody accessibility. Anti-Trp68 antibody also displayed reactivity to mislocalized cytoplasmic TDP-43 in transfected HEK293FT cells (TDP-43^ΔNLS^ and WT TDP-43; Figs. 3*C* and S4). No immunoreactivity was detected in mock transfected and nontransfected HEK293FT cells (Fig. S4), showing the specificity of the anti-Trp68 antibody to misfolded/aggregated TDP-43 molecules. Interestingly, the anti-Trp68 antibody did not recognize TDP-43^ΔNLS^ cytoplasmic aggregates of a mutant Ser68 construct (Fig. 3*C*), suggesting that immunoreactivity to this antibody is a good proxy of Trp-68 solvent exposure or antibody accessibility accompanying misfolding/aggregation. Anti-Trp68 displayed thread-like immunoreactivity against motor neurons in cervical spinal cord sections from two patients who died with sporadic ALS (Fig. 3*D*). The staining had a similar pattern of a commercial anti-phosphorylated TDP-43, demonstrating that misfolding of the WT TDP-43 N-terminal domain occurs in ALS beyond the setting of artificial overexpression in cultured cells. Nuclei in unaffected neuronal and glial cells in these micrographs are not immunoreactive, consistent with the lack of Trp68 solvent exposure in normally folded healthy TDP-43. The above data support the notion that Trp68 is solvent-exposed in pathological TDP-43 aggregates in cell culture *in vitro* and *in vivo* in human sporadic ALS and is available in these conditions to collaborate with natively solvent-exposed Trp172 in RRM1 in the cross-seeding of SOD1 misfolding.

Considering that TDP-43 mislocalization and aggregation occurs in roughly 45% of the cases of frontotemporal dementia (FTD), we were curious to see if the anti-Trp68 antibody would show immunoreactivity in post-mortem cases of this disease (30). To this end, we compared the staining of anti-Trp68 against a commercial anti-phosphoTDP-43 antibody and pan-TDP-43 in the anterior cingulate cortex obtained from patients with confirmed FTD-TDP subtypes A, B, C, and E, imaging both the gray and white matter (Fig. 4). FTD-TDP subtypes are partly characterized by the location, morphology, and extent of the TDP-43–positive inclusions (31, 32). We observed that anti-Trp68 was capable of staining each of the examined FTD-TDP subtypes (Fig. 4). In comparison to commercial the anti-phosphoTDP-43 antibody, anti-Trp68 showed a stronger staining preference for thread-like inclusions (Fig. 4, *A*, *B*, *E*, *F*, *I*, *J*, *M*, and *N*). The anti-phosphoTDP-43 antibody stained extracellular aggregates (Fig. 4*D*) and cytoplasmic granules (Fig. 4, *O* and *P*), whereas anti-Trp68 did not show such staining. This indicates that the Trp68 epitope is either buried or cleaved from the TDP-43 that is the constituent of these inclusions.

### The tryptophan-mediated cross-seeding activity of TDP-43 on SOD1 may constitute a target for ALS therapeutics

The apparent ability of aggregated TDP-43 to kindle misfolding of human WT SOD1, mediated in part by bi-molecular Trp residues as identified above, suggests that pathological TDP-43 possesses the potential to seed the propagated misfolding of human WT SOD1 (33). We have found that propagated misfolding of human WT SOD1 displays cytotoxicity *in vitro* (13), despite the fact that it does not form intracellular aggregates characteristic of mutant SOD1 familial ALS. If seeding of WT SOD1 by TDP-43 occurs in sporadic ALS, manifesting neurotoxicity in addition to TDP-43 toxicity mediated by nuclear depletion and cytoplasmic aggregation and then inhibiting the intermolecular interactions between TDP-43 and SOD1 may represent a target for interventions to slow disease progression. To test this idea, we employed two methods to interfere with the TDP-43-SOD1 cross-seeding in human cell lines and in zebrafish. These methods target the interaction between TDP-43 and SOD1 *via* their respective tryptophans: 1) single-chain intrabodies directed against the RRM1 domain of TDP-43 (34), and 2) 5-fluorouridine, a small molecule that interacts with SOD1 at Trp32 (13, 14, 15, 16, 22).

**Figure 5:**
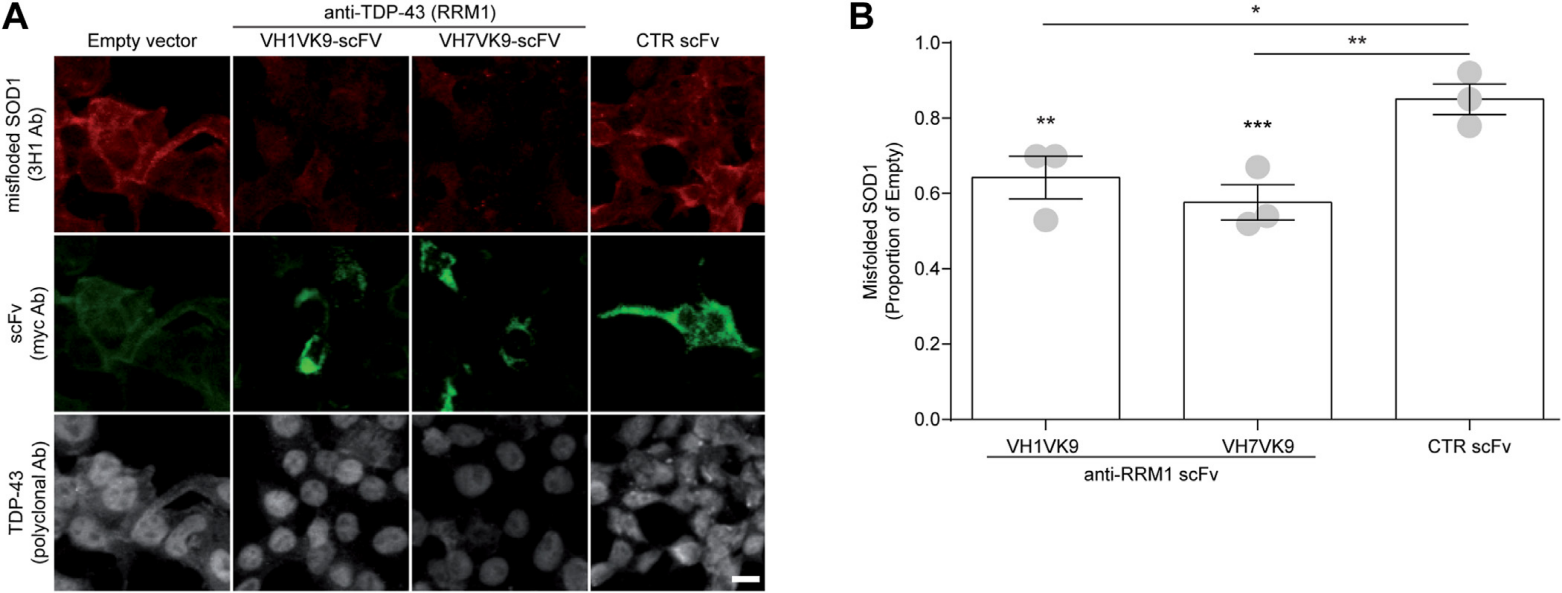
**TDP-43–mediated SOD1 misfolding is attenuated by anti-RRM1 single chain antibodies.***A*, representative image of endogenous SOD1 misfolding (3H1 antibody, *red*) in HEK293 cells co-transfected with WT TDP-43 (*gray*) and anti-RRM1 scFvs (*green*) (VH1Vk9 or VH7Vk9), control scFv (D1.3, anti-chicken lysozyme), or empty vector for scFv. Scale bar represents 10 μm. *B*, quantification of misfolded SOD1. Data are expressed as mean ± SEM (five fields of view were quantified per condition; n = 3 experiments per condition, represented as dots in the bar graph). For each field of view, the integrated density of misfolded SOD1 (3H1) was measured and normalized to the total number of cells. Normalized misfolded SOD1 in scFv-transfected cells was then expressed as fold change compared to cells transfected with the empty vector (no scFv). Statistical significance was established using one-way ANOVA followed by Tukey’s multiple comparison test, ∗*p* < 0.05, ∗∗*p* < 0.01, and ∗∗∗*p* < 0.001 *versus* empty vector (∗) or control scFv antibody (#). RRM, RNA recognition motif; SOD1, superoxide dismutase; TDP, TAR DNA-binding protein.

One of the key Trp residues in TDP-43 required for SOD1 aggregation, Trp172, is located in the RRM1 domain. We hypothesized that blocking this domain would attenuate the seeding of propagated misfolding of SOD1. We applied single chain antibodies (scFv) targeting the RRM1 domain of human TDP-43 (34). These antibodies were previously demonstrated to interact with cytoplasmic TDP-43, reducing its accumulation, and inhibiting NF-kB inflammatory signal generation (34). Here, we cotransfected HEK293 cells with WT TDP-43 and intrabodies against the RRM1 (VH1Vk9 and VH7Vk9) and quantified SOD1 misfolding using 3H1 immunocytochemistry (5, 10). Consistent with our previous findings (5, 35), overexpression of WT TDP-43 triggered WT endogenous SOD1 misfolding (Fig. 5). SOD1 misfolding was significantly reduced in the presence of anti-RRM1 scFv ∼2-fold compared to co-transfection of WT TDP-43 with empty-vector or control scFv (Fig. 5*B*). Together with the above findings, we conclude that RRM1, which contains the natively solvent-exposed Trp172, likely participates in TDP-43–induced kindling of SOD1 misfolding and propagation and may constitute a target for therapeutic blocking of this pathological interaction.

**Figure 6:**
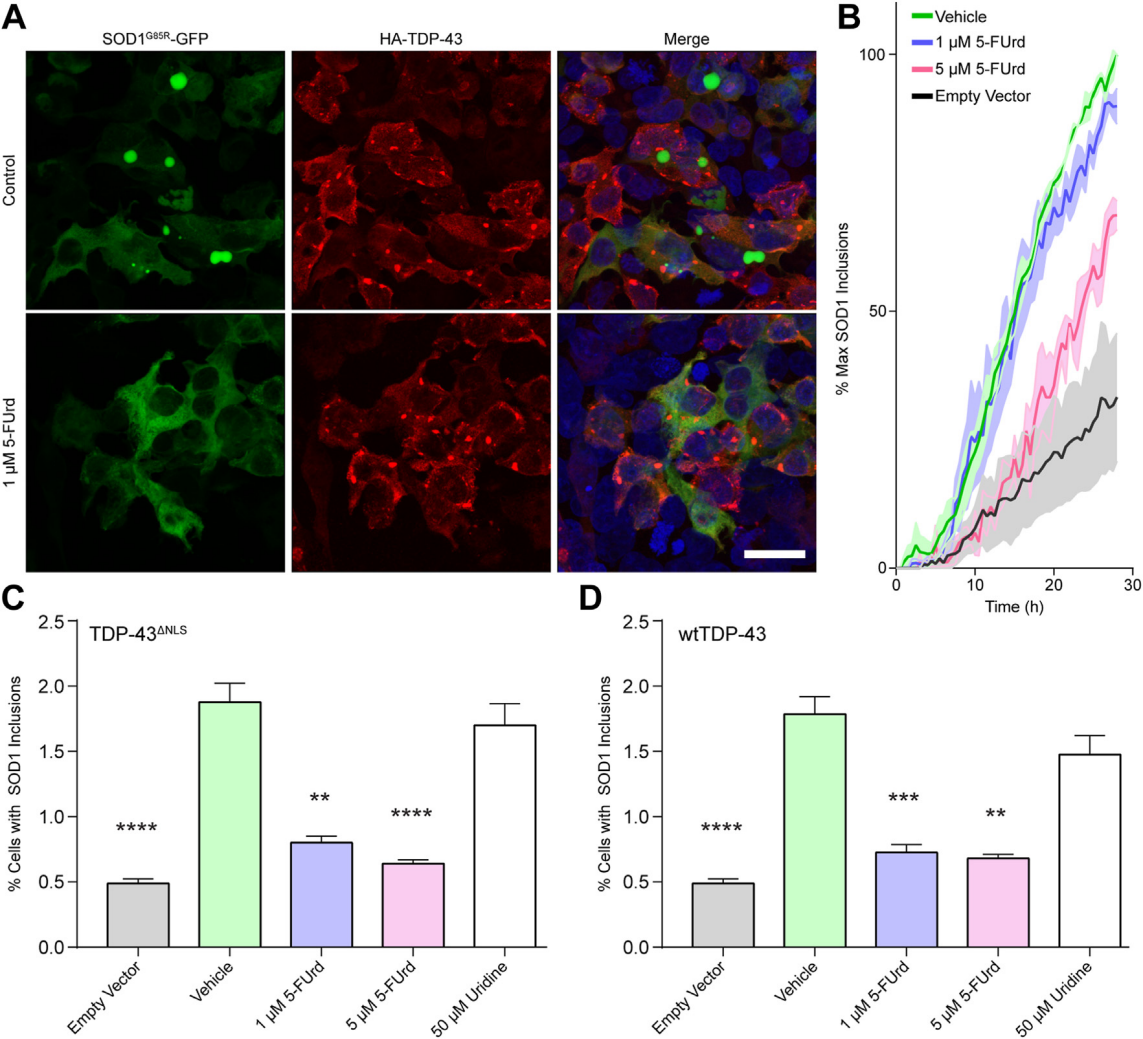
**5-FUrd alleviates cross-seeding of SOD1 by TDP-43.***A*, immunocytochemistry of HEK293FT cells 48 h post co-transfection with TDP-43^ΔNLS^ (*red*) and SOD^G85R^-GFP reporter protein (*green*) in the presence of vehicle (*top*) or 1 μM 5-FUrd (*bottom*), demonstrating a reduction in SOD1 aggregates with 5-FUrd. Scale bar represents 50 μm. *B*, time-lapse live-cell microscopy of SOD^G85R^-GFP cross-seeding by TDP-43^ΔNLS^ in the presence of 1 or 5 μM 5-FUrd shows a reduction in aggregate accumulation proportionate to drug concentration. Images were acquired every 30 min for 30 h. *C* and *D*, flow cytometry of saponin-treated cells co-transfected with the reporter protein with either TDP-43^ΔNLS^ (*C*) or WT TDP-43 (*D*) showing a reduced number of SOD1^G85R^-GFP aggregate containing cells when treated with 5-FUrd. For each experiment, error bars represent SEM of 8 to 13 biological replicates. Statistical significance was determined using one-way ANOVA and Dunnett’s test for multiple comparisons (∗∗*p* < 0.01; ∗∗∗∗*p* < 0.0001). SOD1, superoxide dismutase; TDP, TAR DNA-binding protein.

We next explored if SOD1 itself could also be considered as a therapeutic target. The human SOD1 Trp32 is required for the seeding and propagated misfolding of WT and mutant SOD1 (13, 14) and may thus represent another ALS therapeutic target. Because 5-fluorouridine (5-FUrd) can prevent induction of SOD1 aggregation by mutant SOD1 *via* blocking Trp32 (13, 14, 15), we speculated that this drug might also block TDP-43–induced propagated seeding of SOD1 reporter protein in cells and alleviate axonopathy in zebrafish. 5-FUrd (1–5 μM) significantly inhibited the TDP-43^ΔNLS^–induced aggregation of SOD1^G85R^-GFP in HEK293FT cells (Fig. 6*A*). 5-FUrd did not affect the aggregation status of TDP-43^ΔNLS^, suggesting that 5-FUrd inhibition is specific for SOD1 Trp32. Time-lapse live-cell microscopy revealed that the TDP-43–induced aggregation of SOD1^G85R^-GFP reporter protein is reduced proportionately with increasing concentrations of 5-FUrd (1 and 5 μM, Fig. 6*B*). The rate of SOD1^G85R^-GFP aggregation during the linear aggregate growth-phase (approximately 8–24 h after data acquisition or 24–40 h post co-transfection) apparently decreases with increasing concentrations of 5-FUrd; however, the correlation was not statistically significant (Fig. S5), possibly due to similar outcomes for both drug doses. The efficacy of 5-FUrd in reducing TDP-43–induced SOD1 aggregation was validated independently by quantifying cells with flow cytometry analysis (Fig. 6*C*). A treatment of 5 μM 5-FUrd reduced SOD1^G85R^-GFP aggregation to levels comparable to the control, in which empty vector was used in the place of TDP-43^ΔNLS^. These results support the notion that drugs targeting Trp32 may ameliorate at least some SOD1-mediated neurotoxicity in ALS. Treatment of cells with the nonfluorinated pyrimidine base uridine did not inhibit aggregate formation, even at a much higher concentration of 50 μM (Fig. 6, *C* and *D*). Finally, towards *in vivo* drug application, we considered that the toxicity of 5-FUrd could be mitigated during chemotherapy regimens by co-administering uridine (36). We confirmed that supplementing with uridine does not decrease the ability of 5-FUrd to reduce TDP-43–induced aggregation of SOD1 *in vitro* (Fig. S6).

**Figure 7:**
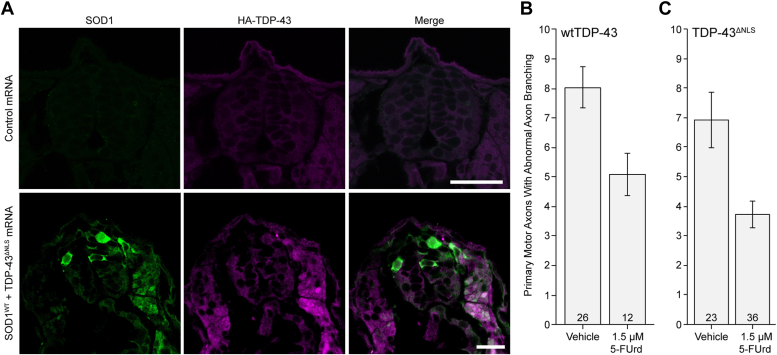
**5-FUrd rescues axonopathy induced by co-expressing human SOD1 and WT TDP-43 in zebrafish.** When human SOD1 and TDP-43 are co-expressed in zebrafish (*A*, exemplar cross-sections of spinal cord), the motor neurons exhibit axonopathy (*B* and *C*), and this axonopathy can be partially but significantly rescued with drugs that block human SOD1 Trp32. Scale bar represents 200 μm. *B*, 5-fluorouridine (5-FUrd, 1.5 μM) rescued axonopathy in embryos injected with SOD1 and WT TDP-43 (*p* = 0.011). *C*, a significant reduction was also observed with 5-FUrd in SOD1 and TDP-43^ΔNLS^-injected embryos (*p* = 0.040). Both vehicle and 5-FUrd solutions contained 5 μM uridine and 0.2% DMSO. Error bars indicate SEM; ∗*p* < 0.05 (Mann-Whitney pairwise comparisons); sample sizes are noted at the base of each bar. SOD1, superoxide dismutase; TDP, TAR DNA-binding protein.

Given our findings above in HEK293FT cells, we tested the capacity of 5-FUrd to rescue SOD1-TDP-43–induced axonopathy in zebrafish (Fig. 7*A*). A low dose of 1.5 μM 5-FUrd (+5 μM uridine) rescued the axonopathy phenotype significantly in SOD1 + WT TDP-43-injected embryos by 36% and in SOD1 + TDP-43^ΔNLS^ embryos by 46% (Fig. 7, *B* and *C*).

## Discussion

While the formation of inclusions incorporating WT TDP-43 is a hallmark of sporadic ALS (4), several studies have identified the presence of misfolded, although typically unaggregated, WT SOD1 in sporadic ALS and non-SOD1 familial amyotrophic lateral sclerosis using an array of SOD1 misfolding-specific antibodies (5, 6, 7, 8, 9, 37, 38). Misfolded, but unaggregated, endogenous WT SOD1 has also been detected in cell cultures overexpressing WT or cytosolically mislocalized mutant TDP-43 (5), which we have replicated in the current manuscript (Fig. 5) in the course of testing TDP-43 RRM1 intrabodies. Once misfolded, this species of WT SOD1 (perhaps oligomeric; (39, 40)) can be transmitted to other populations of naïve cells, thereby triggering additional rounds of induced SOD1 misfolding, which is accompanied by cytotoxicity (35, 41). Previous investigations have yielded little genetic interaction between SOD1 and TDP-43, including a failure of SOD1 mRNA to rescue TDP-43 knockdown- or mutant-induced axonopathy in zebrafish (42, 43, 44, 45, 46, 47). Here, we tested a novel hypothesis in cell culture and zebrafish to demonstrate a pathologic synergy between SOD1 and TDP-43 *in vitro* and *in vivo*, which occurs at a posttranslational level and is mediated by specific solvent-exposed Trp residues in both proteins. We find that overexpression of either mutant TDP-43^ΔNLS^ or WT TDP-43 can cross-seed SOD1^G85R^-GFP reporter protein in HEK293FT cell cultures and that expression of either human WT TDP-43 or human WT SOD1 result in motor axonal toxicity in zebrafish, an effect amplified by their co-expression. We note that our flow cytometry–based assay to detect fluorescent protein inclusions does not take into account transfection efficiency. As such, the seemingly low number of cells containing SOD1 inclusions is likely higher if we measured it in relation to the number of transfected cells. In future, such flow cytometry assays should incorporate markers to identify cotransfected cells. Regardless, we detected that the aggregation of SOD1-G85R-GFP was significantly increased by co-expression with of TDP-43 but not Trpless or Ser68/Ser172 variants.

As in human sporadic ALS, zebrafish TDP-43 toxicity is associated with cytosolic TDP-43, but misfolded human WT SOD1 immunoreactivity is not concentrated into discrete aggregates (Fig. 7*A*), consistent with WT SOD1 misfolding in sporadic ALS. We also show that induced conversion of SOD1 species by TDP-43, in both HEK293FT cells and zebrafish, is mediated by specific tryptophan residues in both proteins, militating for a direct protein–protein interaction. For SOD1, the aggregation of SOD1^G85R^-GFP (in cultured cells) and cross-seeding of human WT SOD1 (in zebrafish) is dependent on solvent-exposed SOD1 Trp32, similar to earlier studies of SOD1-SOD1 propagated misfolding (10, 11, 13, 14, 22). Cross-seeding of WT SOD1 by TDP-43^ΔNLS^ and WT TDP-43, and SOD1^G85R^-GFP aggregation, are apparently dependent on the natively solvent-exposed Trp172 (located in RRM1) and the misfolding/aggregation-induced exposure of Trp68 by loss-of-structure of the TDP-43 NTD. Interestingly, although we have found that exposure of Trp68 due to misfolding/aggregation of cytosolically mislocalized TDP-43 is a requirement for efficient cross-seeding of human SOD1, efficient conversion also requires the collaboration of the additional Trp172 which is natively solvent-exposed. Moreover, the three solvent-exposed Trps in the C-terminal low complexity domain do not substitute for exposure of Trp68, indicating that NTD and RRM1, play a key role in this process which is driven by specific solvent-exposed Trps.

Although a hydrophobic interaction involving the burial of two solvent-exposed Trps between two proteins may not be sufficient to appreciably stabilize a complex (48, 49), a homotypic interaction involving burial of two Trps such as W113, with 200 Å^2^ of exposed surface area each, could result in up to (15 cal/mol⋅Å^2^) (400 Å^2^) ≈6 kcal/mol of free energy, corresponding to a relative Boltzmann probability of p(no interaction)/p(interaction) ≈1/22,000. It is possible that such interactions could bias toward some early intermediate along the misfolding pathway to influence downstream prion-like “strain” characteristics, as has been reported for SOD1 *in vitro* (10, 11, 13) and in mouse and zebrafish models *in vivo* (12, 14). Mutations of Trp32 to either Ser or Phe have both been shown to reduce the aggregation propensity of SOD1 ALS mutants in cultured cells (11, 12, 13), consistent with a role for Trp32 in intracellular misfolding and propagation. Moreover, Trp32 apparently modulates the onset time of clinical paralysis and downstream structural morphology of SOD1 intraneuronal inclusions in SOD1^G85R-YFP^ mice injected with recombinant SOD1 fibrils that possess or lack Trp32 (12).

Pathological activities of mutant or WT TDP-43 are generally thought to include at least two distinct toxicities: (i) loss-of-function of native TDP-43 that is accompanied by nuclear depletion, which includes defective RNA splicing, transport and stabilization, and DNA repair (50, 51) and (ii) gain-of-function from TDP-43 misfolding and cytosolic aggregation (52). These two toxicities may actually be synergistic, including acceleration of nuclear depletion from the recruitment of functional TDP-43 to the cytosolic aggregates by a prion-like mechanism (53). Although misfolded TDP-43 has well-delineated toxicity on its own, including the initiation of ER stress and mitochondrial dysfunction (54, 55), a recently recognized toxic activity of misfolded or aggregated TDP-43 is to trigger the misfolding and dysfunction of other proteins, including nuclear pore proteins and karyopherins (56), proteins involved in mRNA translation (RACK1, DISC1) (57, 58), and proteins implicated in other neurodegenerative diseases (tau, alpha synuclein) (59, 60). Thus, it is perhaps not surprising that SOD1 might also be included in the “pathological interactome” of TDP-43.

Although the toxic prion-like propagation of mutant SOD1 is well recognized, misfolding of human WT SOD1 may be an important early substrate of TDP-43 seeding. Once initiated, human WT SOD1 misfolding can propagate continuously within cells and between cells *in vitro* (10, 61, 62), potentially resulting in the cytotoxic gain-of-function (35) and/or loss-of-function from diminished dismutase activity (63). Recent data has implicated a role for toxic forms of WT SOD1 secreted from sporadic ALS astrocytes (64) and detected in sporadic ALS cerebrospinal fluid (65). Interestingly, it has been reported that misfolded mutant SOD1 acquires its own pathological interactome, including G3BP1, α-synuclein, and amyloid-β (17, 66, 67). The concept that SOD1 misfolding and toxicity is downstream of TDP-43 is consistent with reports that the emergence of the sporadic ALS syndrome may be preceded by six mathematically defined preclinical “steps” (68) but that familial amyotrophic lateral sclerosis mutations in TDP-43 are best captured by a model comprising four steps and that SOD1 mutations apparently require only two steps (69), suggesting that WT SOD1 may be downstream of other events in sporadic ALS pathogenesis, such as TDP-43 aggregation.

If misfolded WT SOD1 possesses toxic activities beyond those inherent in TDP-43 loss- or gain-of-function, the relevance of the identified set of Trp residues to the development of SOD1 and TDP-43 toxicity at the whole organism level is a compelling avenue for further exploration. We explored the therapeutic relevance of Trp32 in this TDP-43-SOD1 paradigm using 5-FUrd, a candidate drug predicted to block Trp32 at the molecular surface of human SOD1 protein (13, 14, 15, 70). Since 5-FUrd successfully reduces SOD1 aggregation in cell culture and reduces motor neuron toxicity in zebrafish, it may be an attractive candidate for further investigation, not only in mutant SOD1-familial ALS but sporadic ALS as well. It is also possible that partial knockdown of human WT SOD1 through antisense oligonucleotides or RNA-based therapies might be tested in sporadic ALS, beyond the SOD1-mutant familial ALS for which these gene therapy modalities have recently demonstrated efficacy (71, 72).

## Experimental procedures

For more comprehensive experimental procedures, please see the supplementary information.

### Imaging and quantification of SOD1 inclusions using microscopy

Time-lapse live-cell imaging was carried out on HEK293FT cells cultured in 8-well microscope chambers. Quantification of SOD1^G85R^-GFP reporter protein aggregation was performed as previously described (4). Briefly, the chamber was placed into a microscope-mounted incubation system to maintain ideal cell culture parameters (humidified, 5% CO_2_ at 37 °C). Image acquisition began 16 h post-transfection (to allow protein expression) using an A-Plan 10 × objective with a numerical aperture of 0.25 mounted on an inverted Axio Observer Z1 microscope equipped with an AxioCam HighRes camera (Carl Zeiss AG) and a motorized stage. Images were acquired at 1024 × 1024 pixel resolution (3 × 3 grid – stitched post-acquisition) every 30 min. Images were exported as high resolution JPG files.

Quantifying induced aggregation of SOD1^G85R^-GFP reporter protein utilized a custom algorithm developed in ImageJ that reliably measured inclusions based on the area that inclusions normalized to total expressed GFP (4, 5). Briefly, images are converted into 8 bit images, local background is subtracted *via* rolling-ball method, threshold is set based on background soluble reporter protein, and aggregate size is quantified (minimum size of 5 pixels to avoid noise; maximum size was set to one third of an average cell size). Total fluorescence area of all fluorescent reporter protein is also quantified. The percentage of aggregation is reported as a ratio of area of inclusions divided by the total area of fluorescence. Aggregate abundance was reported as a percentage of the aggregate abundance observed at various experimental time-points or end-stage of the experiment when using Trp-containing construct. Statistical significance was determined using one-way ANOVA and Dunnett’s test for multiple comparisons, calculated in GraphPad Prism 7 (https://www.graphpad.com/).

### Immunohistochemistry on paraffin-embedded human tissue

Brain samples were obtained from The Netherlands Brain Bank, Netherlands Institute for Neuroscience. All donors gave written informed consent for a brain autopsy and the use of the material and clinical information for research purposes and the principles of the Declaration of Helsinki were followed. Dementia status at death was determined on the basis of clinical information available during the last year of life and neuropathological diagnosis using (immuno)histochemical stainings (H&E, Bodian and/or Gallyas silver stainings, and immunohistochemical stainings for Amyloid beta, tau, alpha-synuclein, TDP43, and p62). Tissue was fixed in formalin for 1 month. Analysis of formalin-fixed, paraffin-embedded tissue from different parts of the brain was performed.

FTD tissue was selected if the donor had a clinical history of FTD and TDP43 pathology. Briefly, donors were classified as type A when inclusions and short threads were observed to be predominantly in the superficial layers. If mainly cytoplasmic inclusions were seen with no preference for layers, type B was denoted. If long threads were seen predominantly in the superficial layers, type C was characterized. Type D was given if frequent lentiform intranuclear inclusions were seen due to a mutation in valosin-containing protein, which is very rare. Finally, if the anterior cingulate gyrus showed a strong granular intracellular and extracellular spread with no intracellular inclusions, type E was considered. For this project, we included one type A, one type B, one type C, and one type E donor and included the anterior cingulate gyrus for analysis.

To confirm the specificity of binding, one neurologically healthy control donor was included that was confirmed to be negative for aggregated TDP43. We also included two FTD donors with frozen sections and two donors with a clinical diagnosis of ALS and TDP43 pathology to test binding affinity in ALS tissue.

Cervical spinal cord sections (8 μm thick) from formalin-fixed paraffin embedded issue were mounted on Superfrost plus tissue slides and dried overnight at 37 °C. Sections were deparaffinized and subsequently immersed in 0.3% H_2_O_2_ in methanol for 30 min to quench endogenous peroxidase activity.

The slides were subjected to heat pretreatment with Tris-EDTA and incubated overnight at 4 °C in 0.1 μg/ml rabbit anti-Trp68 diluted in a purchased antibody diluent. Omission of the primary antibodies served as a negative control. A section was incubated with 1:8000 rabbit phospho-TDP43 antibody and one with 1:6000 pan-TDP-43 (Abnova) as a positive control. Secondary EnVisonTM HRP goat anti-rabbit/mouse antibody incubation was for 30 min at room temperature. 3,3-Diaminobenzidine from the same kit was used a chromogen for 10 min. Sections were counterstained with hematoxylin to visualize the nuclei of the cells, dehydrated, and mounted using Quick-D mounting medium.

## Ethics approval and consent to participate

Use of zebrafish for this study was approved by the Animal Care and Use Committee: BioSciences at the University of Alberta under protocol AUP00000077, under the auspices of the Canadian Council on Animal Care. Adult zebrafish were maintained and bred according to standard procedures, including housing in brackish water (1250 ± 50 μS) at 28.5 °C.

Brain samples were obtained from The Netherlands Brain Bank, Netherlands Institute for Neuroscience (Amsterdam, The Netherlands). All donors gave written informed consent for a brain autopsy and the use of the material and clinical information for research purposes. All human research was carried out according to the principles of the Declaration of Helsinki.

## Data availability

All materials and data are available upon reasonable request to the corresponding authors.

## Supporting information

This article contains supporting information (13, 14, 22, 24, 29, 34, 73, 74, 75, 76, 77).

## Conflict of interest

J.-P. J. and S. P. are the owners of a patent US 15/532,909 titled “TDP-43-binding polypeptides useful for the treatment of neurodegenerative diseases”. J.-P. J. is the chief scientific officer of Imstar Therapeutics. The 3H1 misfolded SOD1 antibody used in this manuscript is owned by the University of British Columbia and licensed by ProMIS Neurosciences. N. R. C. is the Chief Scientific Officer of ProMIS Neurosciences. S. S. P. and N. R. C. have received consultation compensation from ProMIS and possess ProMIS stock and stock options. All other authors declare that they have no conflicts of interest with the contents of this article.
