# Supplementary figures and images for: Tryptophan residues in TDP-43 and SOD1 modulate the cross-seeding and toxicity of SOD1
Source: J Biol Chem. 2024 Mar 22;300(5):107207. doi: 10.1016/j.jbc.2024.107207 (PMC11087967; doi:10.1016/j.jbc.2024.107207)

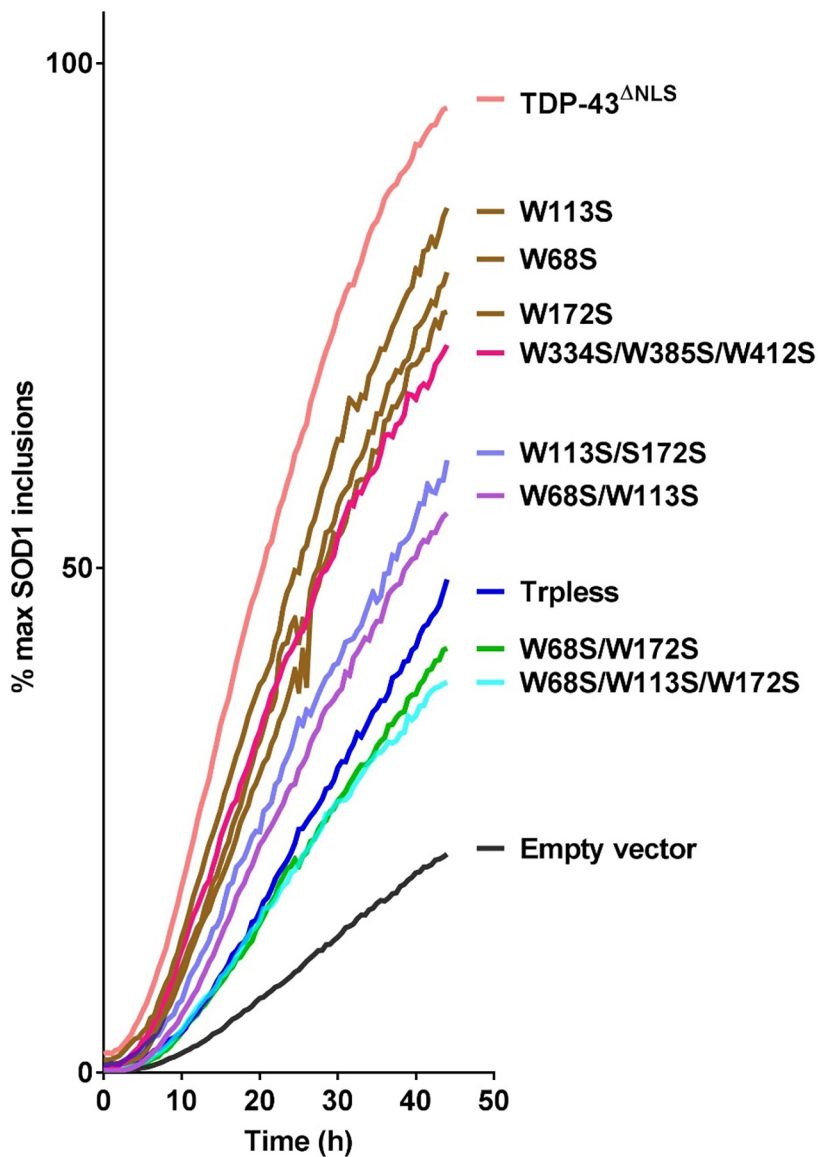

Supplement: Supporting Figure S1 [file mmc1.pdf]

**A**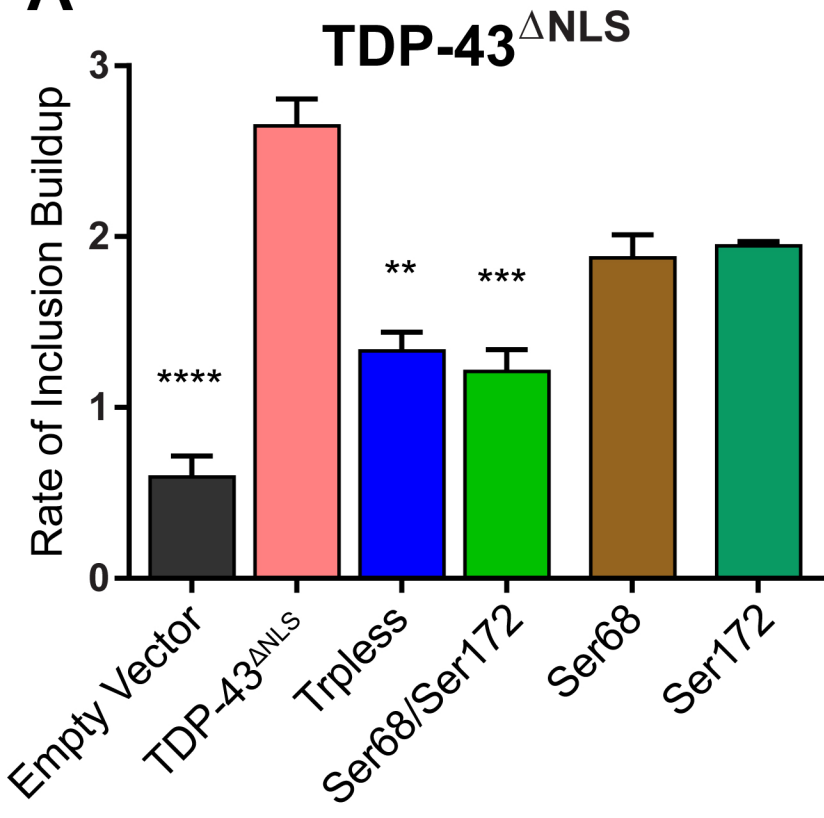**B**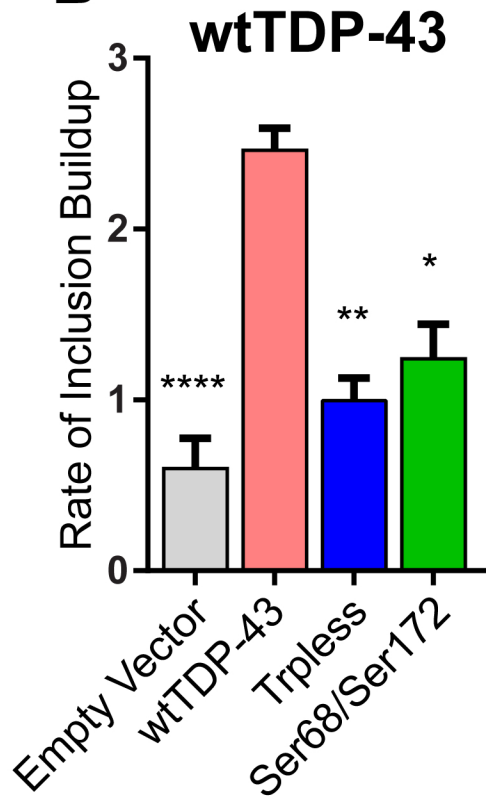

Supplement: Supporting Figure S2 [file mmc2.pdf]

**A**

Native gel

SDS gel

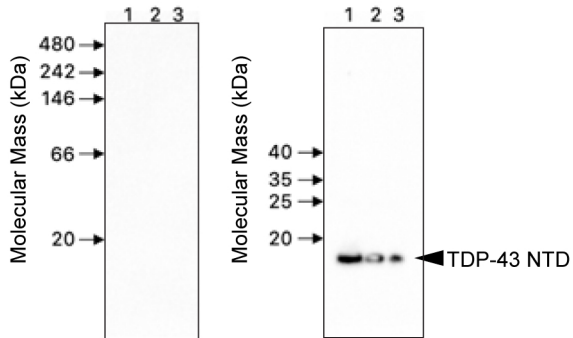**B**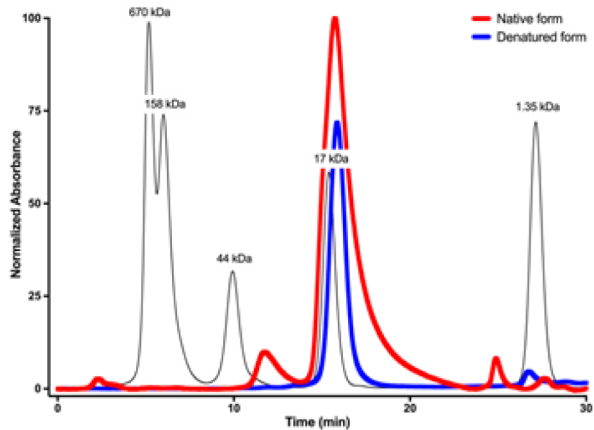

Supplement: Supporting Figure S3 [file mmc3.pdf]

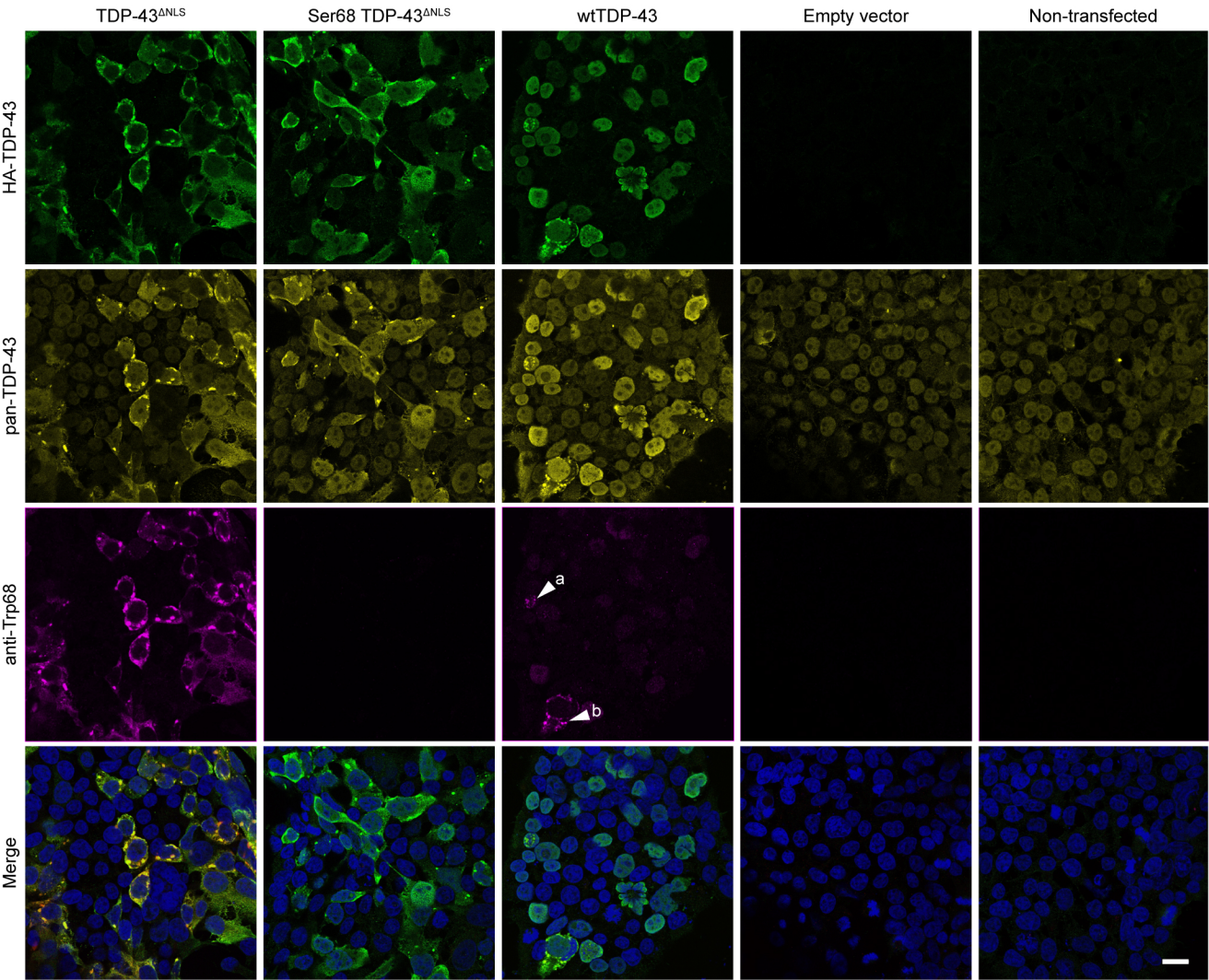

Supplement: Supporting Figure S4 [file mmc4.pdf]

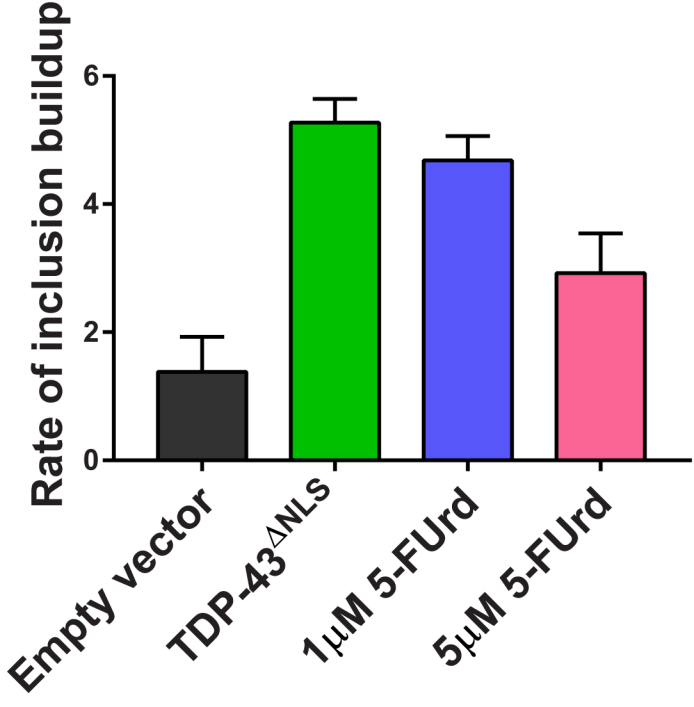

Supplement: Supporting Figure S5 [file mmc5.pdf]

**A**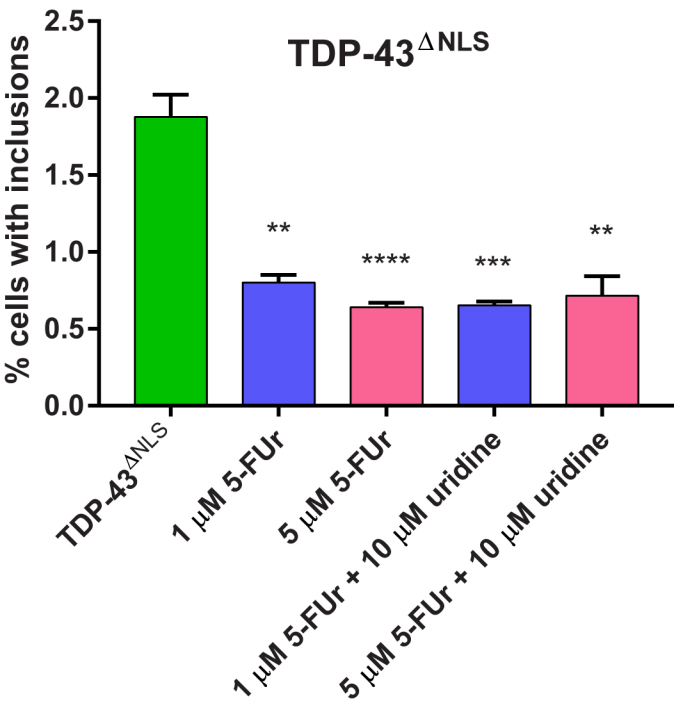**B**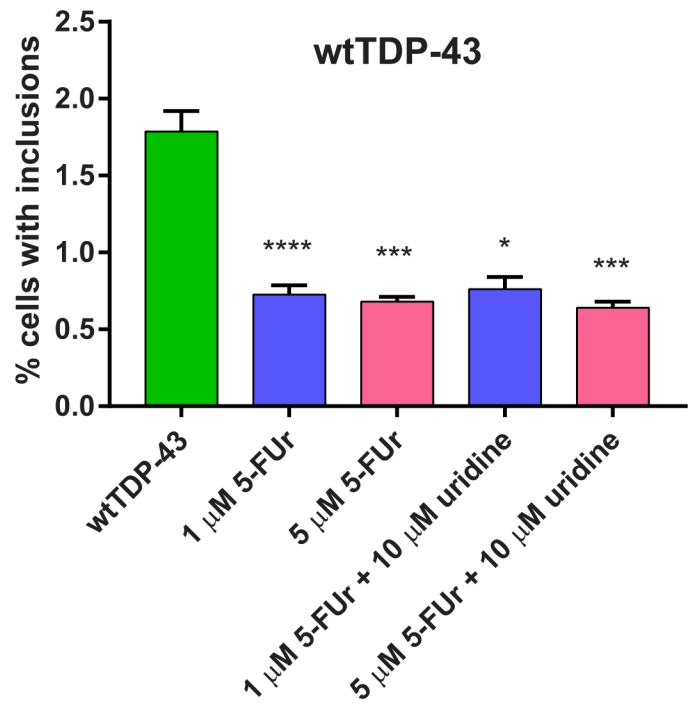

Supplement: Supporting Figure S6 [file mmc6.pdf]
