# Supplementary material for: Tryptophan residues in TDP-43 and SOD1 modulate the cross-seeding and toxicity of SOD1
Source: J Biol Chem. 2024 Mar 22;300(5):107207. doi: 10.1016/j.jbc.2024.107207 (PMC11087967; doi:10.1016/j.jbc.2024.107207)
Supplement: Supporting Information [file mmc7.docx]

Supplementary Information for

**Tryptophan residues in TDP-43 and SOD1 modulate the cross-seeding and toxicity of SOD1**

Edward Pokrishevsky, Michèle G. DuVal, Luke McAlary, Sarah Louadi, Silvia Pozzi, Andrei Roman, Steven S Plotkin, Anke Dijkstra, Jean-Pierre Julien, W. Ted Allison, Neil R. Cashman

**Corresponding Authors**: W. Ted Allison and Neil R. Cashman

**Email:** ted.allison@ualberta.ca

**Email:** Neil.Cashman@vch.ca

**This PDF file includes:**

Supplementary Methods and Materials

Supplementary material references

**Other supplementary materials for this manuscript include the following:**

**Supplementary Information Text**

**Extended Methods and Materials.**

***Animal Ethics Statement***

Use of zebrafish for this study was approved by the Animal Care and Use Committee: BioSciences at the University of Alberta under protocol AUP00000077, under the auspices of the Canadian Council on Animal Care. Adult zebrafish were maintained and bred according to standard procedures, including housing in brackish water (1250 ± 50 µS) at 28.5 °C.

***Materials***

Brain samples were obtained from The Netherlands Brain Bank, Netherlands Institute for Neuroscience (Amsterdam, The Netherlands). Both donors had a clinical diagnosis of sporadic ALS with TDP-pathology. They gave written informed consent for CNS autopsy and the use of the material and clinical information for research purposes.

SOD1^G85R^-GFP plasmid was the kind gift from Professor Elizabeth Fisher (University College London, UK) (Addgene: 26410) (73), and a plasmid containing human wild-type TDP-43-FLAG was kindly provided by Dr. M. Urushitani (Shiga University of Medical Science, Japan). A plasmid for expression and purification of the N-terminal domain of TDP-43 (residues 1-80) (74) was generously provided by Dr. Nicolas L. Fawzi (Brown University, USA).

Other materials were purchased from the following companies: Human embryonic kidney cells (HEK293FT and HEK293; ATCC, Manassas, VA), L-glutamine (#25030081,Thermo Fisher, USA), Penicillin-Streptomycin (#15140122, Thermo Fisher, USA), Lipofectamine LTX (#15338100, Thermo Fisher), JetPrime transfection reagent (Polyplus, France), Expand High Fidelity PCR System (#11759078001, Roche, Switzerland) rabbit anti HA-tag antibody (#ab9110, Abcam, USA), chicken anti-HA tag antibody (#ab9111, Abcam, USA), mouse anti-TDP-43 antibody (#60019-2-Ig, Proteintech, USA), mouse anti-human TDP-43 (#H00023435-M01, Abnova, Taiwan), mouse anti-misfolded SOD1 monoclonal antibody 3H1 generated by Cashman lab (29), rabbit anti-TDP-43 C-Terminal antibody (#12892-1AP, Proteintech, USA), rabbit anti-SOD1-100 antibody (#ADI-SOD-100, Enzo Life Sciences), mouse anti-HA antibody (#ab130275, Abcam), rabbit anti-phospho S409/410 TDP43 antibody (Tip-PTD-Mo1, Cosmo Bio, Japan), Bis-benzimide H33342 trihydrochloride (#H3570, Thermo Fisher, Canada), Fluoromount-G (#00-4958-02, SouthernBiotech, USA). Secondary antibodies conjugated to fluorophores that were used for immunocytochemistry (Alexa-Fluor) were raised in goat and purchased from Thermo Fisher, Canada. Trp68 rabbit polyclonal antibodies were custom generated and affinity purified against the TDP-43 amino acid sequence _65_DAGWGNL_71_ by GenScript (Piscataway, NJ).

For immunohistochemistry, we used a kit containing both the HRP labelled goat anti-rabbit/mouse secondary antibody and the 3,3-Diaminobenzidine, called REAL EnVision Detection system (#K5007, Dako, Denmark), and we used a universal antibody dilution buffer whenever the buffer composition is not specified (#U3510-100ML, Sigma, USA). For mounting, we used Quick-D Mounting medium (#7281, Klinipath, Netherlands). Mouse anti-myc 488 conjugated was purchased from Millipore (#16-308). Superfrost plus tissue slides (Menzel-Gläser, Germany), FastDigest NotI restriction enzyme (#FD0593, Thermo Fisher, Canada), Ambion mMESSAGE SP6 transcription kit (#AM1340, Thermo Fisher, Canada), Clear Frozen Section Compound (#95057-838, VWR), Superfrost Plus microscope slides (#12-550-15, Fisher Scientific).

Microscope objectives: 63× objective on the Leica TCS SP8 microscope (#506350, Leica, Germany), A-Plan 10× objective, (#421040-9900-000, Carl Zeiss AG, Germany) equipped with an AxioCam HighRes camera (Carl Zeiss AG, Germany).

For Electrophoresis and Immunoblotting, we purchased pre-cast NativePAGE™ 4–12% Bis-Tris gels and NuPAGE™ 4-12% Bis-Tris Protein Gels, 1.0 mm (#NP0303BOX & NP0321BOX respectively, Thermo Fisher Scientific, USA), NativePAGE™ Sample Buffer (#BN2003, Thermo Fisher Scientific, USA), NuPAGE™ LDS Sample Buffer (# NP0007, Thermo Fisher Scientific, USA), 0.45 µm PVDF Transfer membrane (#88518, Thermo Fisher Scientific, USA), XCell II™ Blot Module (#EI9051,Thermo Fisher Scientific, USA). Amersham ECL HRP-conjugated secondary donkey anti-rabbit antibody (#NA93V, GE Healthcare Life Sciences, USA). SuperSignal™ West Femto Maximum Sensitivity Substrate (34094, Thermo Fisher Scientific, USA) and the ChemiDoc MP Imaging system (Biorad,USA) were used for detection.

For Size Exclusion Chromatography, we used Superdex 75 (10/300) FPLC column (#17517401, GE Healthcare Life Sciences, USA)

***Transfections***

HEK293FT and HEK293 cells were cultured in complete Dulbecco’s Modified Eagle Medium (DMEM) containing 10% FBS, 10 U/ml penicillin, 10 U/ml streptomycin and 2 mM L-glutamine.

Systematic substitution of tryptophan residues in wtTDP-43 and TDP-43^ΔNLS^ was performed using site-directed mutagenesis. All of our TDP-43 constructs are HA-tagged or Flag-tagged to distinguish between endogenous and exogenous protein.

Pre-plated cells were co-transfected with the reporter protein (SOD1^G85R^-GFP), and one of the TDP-43 constructs at a ratio of 1:5, respectively, using Lipofectamine LTX according to manufacturer’s instructions. For the quantification of TDP-43-induced SOD1 misfolding, cells were co-transfected with HA-tagged wild-type TDP-43 and scFv antibodies.

For measurement of TDP-43-induced SOD1 misfolding, plasmid containing human wild type TDP-43-Flag was co-transfected with the pscFv9 plasmid encoding for VH1Vk9 (34), VH7Vk9 anti-RRM1 scFv antibodies, D1.3 scFv anti-chicken lysozyme scFv antibody, or pscFv9 empty plasmid. JetPrime transfection reagent was used according to manufacturer’s instructions. Mouse mAb 3H1 immunocytochemistry was used to quantify the conversion of wild-type endogenous human SOD1 to a misfolded form.

Cells were then incubated for 48 h in a 37 ˚C humidified incubator supplemented with 5 % CO_2_. In some instances, cells were treated with 5’-fluorouridine (5-FUrd). The drug was dissolved in DMEM media under sterile conditions and added at the indicated doses 4 h following the transfection of cells (13).

***Quantification of SOD1 Inclusions using flow cytometry***

The abundance of cells with induced aggregation of SOD1^G85R^-GFP reporter protein was determined using flow cytometry (13, 22). In order to identify only those cells that not only express SOD1^G85R^-GFP, but where this report protein is indeed aggregated, we permeabilized the cells using 0.03% saponin in ice-cold PBS for 10 minutes to allow soluble SOD1^G85R^-GFP to leak out of cells (13, 22). Cells were then washed once using cold PBS and were stored on ice until analysis on LSRII (BD Biosciences, USA). Data analysis was performed using FlowJo.

***Immunocytochemistry***

HEK293FT cells were seeded at a density of 6.3 × 10^5^ cells/cm^2^ on Poly-D-Lysine coated glass cover-slips (#1.5 thickness) in a 24 well plate prior to transfection. 48 h post-transfection, cells were washed twice with ice-cold PBS and fixed in 4% paraformaldehyde (in PBS, pH 7.4) for 15 min at room temperature. Fixed cells were washed once with PBS, permeabilized for 10 min using PBSTx (0.3% Triton X-100 in PBS), and blocked for 30 min with blocking buffer (10% normal goat serum in PBS, filtered). Cells were incubated with primary antibodies diluted in incubation buffer (10% normal goat serum in PBS, filtered) to the following concentrations: 1 µg/ml for both chicken and rabbit anti-HA antibodies, 0.5 µg/ml rabbit anti-Trp68 for 1 h at room temperature. Cells were washed twice in PBS, and incubated with the appropriate secondary antibody (Alexa-fluor 488 anti-mouse, Alexa-fluor 568 anti-chicken, Alexa-fluor 647 anti-rabbit) diluted 1:1000 in blocking solution for 1 h at room temperature in the dark. Cells were then washed with PBS, and DNA was counterstained using 2 µg/ml Hoechst 33342 for 5 min. Following two final washes the cells were mounted on a glass slide in a drop of Fluoromount-G. Confocal images were captured using Leica TCS SP8 microscope on the oil-immersion 63× objective, with a numerical aperture of 1.4 using the LAS-X software (Leica, Germany) at 2048 × 2048 pixel resolution. Images were acquired at the same settings, brightness and contrast were enhanced uniformly across treatment groups for clarity where indicated.

***Quantification of TDP-43-induced SOD1 misfolding***

HEK293 cells were co-transfected with wild-type TDP-43 and scFv antibodies and stained as indicated above with 2 ug/mL 3H1 anti-misfolded SOD1 mouse mAb and 0.4 ug/mL anti-TDP43 C-Terminal antibodies. After incubation of the slides with the appropriate secondary antibodies, scFv antibodies were detected with incubation with mouse anti-myc 488 conjugated diluted 1:500 in PBS for 2 h.

Images were acquired using confocal microscope BX-61 Virtual Stage (Olympus) with a Z-stack and analyzed with ImageJ software. To quantify SOD1 misfolding, the total integrated density was measured for the 3H1 antibody signal in each picture after adjusting threshold to discard signal coming from the empty coverslip. Intensity was then normalized to the total number of cells defined as those positive for nuclear TDP-43 signal. Statistical analysis was a one-way ANOVA followed by Tukey’s multiple comparison test, performed in Prism 5.0 (GraphPad, La Jolla, CA, USA). A *p*-value lower than 0.05 was considered significant.

***Expression of human SOD1 and TDP-43 in zebrafish***

The human SOD1 and TDP-43 genes, including versions with variations indicated, were cloned using Gateway recombination into the pCS2+ expression vector construct for mRNA synthesis (pCS2+.wtSOD1.pA, pCS2+.SOD1^Trp32S^.pA, pCS2+.HA-wtTDP-43.pA, pCS2+.HA-TDP-43^∆NLS^.pA, pCS2+.HA-TDP-43^∆NLS-Trpless^.pA, pCS2+.HA-TDP-43^W68,W113S^.pA, and pCS2+.HA-TDP-43^W68,W172S^.pA). mRNA production was done using FastDigest NotI restriction enzyme and the Ambion mMESSAGE SP6 transcription kit. mRNA was co-injected with 100 pg mCherry mRNA into 1-2-cell stage embryos from *mnx1:GFP* transgenics (ZFIN ID: ZDB-ALT-051025-4) crossed to wild type AB fish. Embryos were screened at 24 h post-fertilization (hpf) for mCherry fluorescence indicating successful injection, and any embryos with a disrupted body axis or other overt defects were excluded from analysis.

Effective dosages of SOD1 or TDP-43 mRNA were determined empirically, with previous publications as guides (14), to robustly induce a measurable phenotype above background (control mRNA) levels, but below maximum levels of axonopathy, and was established at 900 pg. To harmonize amount of mRNA injected between various groups, total mRNA dose was made constant by top-up with innocuous Tol2 transposase mRNA, for a total 1900 pg mRNA per embryo. A control group was utilized to account for injection of exogenous mRNA; the mRNA control group dose consisted of 1800 pg of Tol2 transposase mRNA and 100 pg mCherry mRNA.

***Assessment of axonopathy***

Embryos were raised to 36 hpf, fixed briefly in 4% formaldehyde, and assessed for axonopathy via GFP expression in the primary motor neurons by an observer blinded to the treatments. Dysmorphic embryos were not assessed. The axonopathy phenotype is an established robust assay for abnormal development/maintenance of the primary motor neurons caused by overexpression of wildtype and mutant SOD1, TDP-43, and other genes associated with human neuromuscular disease (24). Across this variety of diseases, intensity of the axonopathy phenotype is strongly correlated with disease severity. Briefly, primary motor axons were scored as abnormal if they exhibited branching dorsal of the notochord’s ventral boundary; total counts of abnormal axons were recorded per embryo (Fig. 2 A). Statistical analysis was performed using Kruskall-Wallis ANOVA with post-hoc Mann Whitney pairwise comparisons in Stata/SE 14.1 for Mac (2015, StataCorp). In some instances, muscle fibers were visualized by staining with Alexa Fluor-555-tagged phalloidin for 1 h. Embryos were subsequently mounted on slides using 1.5% low-melting point agarose and imaged on a Zeiss LSM 700 confocal laser microscope with Zen 2010 software (Carl Zeiss Imaging).

***Drug-based rescue using 5’-fluorouridine in zebrafish***

The effective dosage of 5-fluorouridine (5-FUrd) was determined by establishing a dose-response curve for survival and axonopathy in zebrafish. Embryo media was replaced with media containing either drug or vehicle control when embryos reached 12 hpf. 5-FUrd treatment media contained 1.5 μM 5-FUrd, 5 μM uridine, and 0.2% DMSO, whereas vehicle control media contained 5 μM uridine and 0.2% DMSO. Embryos were kept in drug media until fixation in 4% formaldehyde at 36 hpf.

***Zebrafish Immunohistochemistry***

For labeling to detect human SOD1 and HA-TDP-43^ΔNLS^ in zebrafish, embryos (without GFP transgenes) were fixed at 30 hpf and cryopreserved as previously described using step-wise sucrose/0.1 M PO_4_ washes and freezing in sucrose/PO_4_ mixed with Clear Frozen Section Compound overnight at -80 °C (24). 10 μm cryosections were mounted on Superfrost Plus microscope slides, allowed to air-dry, and frozen at -80 °C overnight. Slides were then thawed, incubated in 10% normal goat serum/PBSTw, and incubated in primary antibodies overnight: 1:500 rabbit anti-SOD1-100 (Enzo Life Sciences, ADI-SOD-100) and 1:100 mouse anti-HA (Abcam, ab130275). After washes with PBSTw, slides were incubated in Alexa-fluor 488 anti-rabbit and Alexa-fluor 647 anti-mouse antibodies overnight. Following final washes, slides were imaged on a Zeiss LSM 700 confocal laser microscope with Zen 2010 software (Carl Zeiss Imaging).

***Purification of TDP43 NTD***

Protein was expressed in *Escherichia coli* and purified as described previously (74). Purified TDP-43 NTD was dialyzed against PBS, concentrated to 0.5 mg/ml and stored at −80 °C

***Electrophoresis using SDS/Native gels and Immunoblotting***

Native-PAGE was carried out using the Novex Bis-Tris system according to the manufacturer’s specifications. Protein samples were mixed with the NativePAGE™ Sample Buffer. Pre-cast NativePAGE™ 4–12% Bis-Tris gel was run at 4 °C at 150 V constant for 60 min, then at 250 V for the 30 min.

SDS-PAGE was carried out using the Novex Bis-Tris system according to the manufacturer’s specifications. Protein samples were mixed with the NuPAGE™ LDS Sample Buffer. Pre-cast NuPAGE™ 4–12% Bis-Tris gel was run at RT at 200 V constant for 35 min.

Proteins were blotted onto 0.45µm PVDF membranes using the XCell II Blot Module following the manufacturer's protocol. Blots were blocked in 5% milk powder in 0.0.2% Tween 20 Tris Buffer Saline, and then were incubated with 0.5 µg/mL Trp68 antibody overnight at 4 °C. For detection on the ChemiDoc MP, a donkey anti-Rabbit IgG HRP-labelled secondary antibodies was used. The SuperSignal West Femto substrate was used according to the manufacturer’s instructions.

***Size Exclusion Chromatography***

Analytical gel-filtration of TDP43 NTD forms was performed using high performance liquid chromatography on Superdex 75 (10/300) HPLC column. TDP-43 NTD (0.5 mg/ml) was denatured by incubation in 6 M Guanidine-HCl, 50 mM Tris-HCl buffer (pH 7.5.), 150 mM NaCl, 20 mM DTT, for 10 min at 37 °C. The obtained protein sample (100 μl) was loaded onto the column pre-equilibrated with the same buffer and eluted at 0.5 ml/min. Native form was loaded onto the column pre-equilibrated with the 1x PBS buffer containing 5 mM DTT and eluted at 0.5 ml/min.

***Computational Protein Modelling***

Representative structures were obtained as the top-ranked structures using 3D-Jury ranking with MaxSub comparison between structures and threshold RMSD of 2 (75). The MaxCluster program was used to obtain the 3D-Jury ranking (76). Native conformational ensembles of the N-terminal domain and RRM1 domain of TDP43 were obtained from equilibrium molecular dynamics simulations using GROMACS 4.5 and 5.0 respectively (77). Apo structures without nucleic acid or other ligand were used to calculate the SASA of Tryptophans in a non-redundant database of 27,015 structures taken from the PDB, as described in (29).

***Statistical Analysis***

Statistics were performed as described above in figure legends.

**Supplementary Figures Legends**

**Supplementary Figure 1: Tryptophan residues at position 68 and 172 in TDP-43^ΔNLS^ are most critical for aggregation of mutant SOD1- based reporter protein.** Time-course algorithm count of induced SOD1 aggregates in HEK293FT cells from 24 to 72 h post co-transfection with the indicated TDP-43^ΔNLS^ and its variants. Images were acquired every 30 minutes. Time-point h = 0 corresponds to the beginning of imaging, which occurred approximately 16 h post-transfection. The number of inclusions at every time point is expressed as a percentage of the final inclusions of TDP-43^ΔNLS^ in the biological repeat. Error bars were removed for clarity reasons. Each curve represents 3-18 independent biological repeats.

**Supplementary Figure 2: The rate of SOD1 inclusion formation is dependent on the presence of tryptophan residues in TDP-43**. Cells co-transfected with SOD1 and TDP-43 show a rapid linear growth of SOD1 inclusions, whereas those with TDP-43 variants lacking all (Trpless) or two key tryptophans (Trp68Ser/Trp172Ser) exhibited a much slower linear growth. The rate of inclusion build-up per hour, plotted here, is based on the linear growth phase of the SOD1 inclusions in the presence of TDP-43^ΔNLS^ (**A**) or wild-type TDP-43 (**B**) based constructs and was quantified from 10-35 h after data acquisition or 26-51 h post co-transfection. Statistical significance was determined using one-way ANOVA and Dunnett’s test for multiple comparisons (* p < 0.05; ** p <  0.01; *** p < 0.001; **** p  <  0.0001).

**Supplementary Figure 3: Trp68 is not accessible to an anti-Trp68 antibody unless the TDP-43 N-terminal domain is denatured. (A)** The anti-Trp68 antibody used in immunoblot analysis of native- and SDS-PAGE gels shows reactivity only to denatured TDP-43 NTD. Lanes 1, 2, 3 contained 0.6, 0.3 and 0.15 µg protein per lane, respectively. **(B)** SEC fractionation chromatogram of TDP-43 NTD in native form (red line) and denatured in 6 M guanidinium chloride solution (blue line). The protein remains monomeric in both conditions. MW markers are superimposed for reference (black line).

**Supplementary Figure 4: Trp68 is exposed in aberrant TDP-43- containing cytoplasmic and nuclear aggregates**. The rabbit anti-Trp68 antibody (red) was tested for reactivity and specificity in cells transfected with different TDP-43 constructs. A mouse pan-TDP-43 antibody against the C-terminal domain (Proteintech, USA) and a chicken anti-HA-tag antibody (Abcam, USA) were used to test co-localization with TDP-43 (yellow) and over-expressed TDP-43 (green) respectively. The anti-Trp68 antibody specifically recognizes mislocalized cytoplasmic TDP-43 aggregates in TDP-43^ΔNLS^-transfected cells aberrant nuclear TDP-43 aggregates that form when TDP-43 is overexpressed. Trp68 is also exposed in cytoplasmic aggregates that form when TDP-43 is overexpressed in wild-type TDP-43 transfected cells, and these aggregates are recognized by the specific antibody. However, Trp68 does not stain non-aggregated nuclear TDP-43 nor does it recognize cytoplasmic TDP-43 aggregates lacking Trp68. No background staining was seen in mock transfected and non-transfected cells. Scale bar: 20 µm. The authors declare image reuse of panels from this figure in main text figure 3 which are zoomed in to make interpretation of the finer details of the images easier.

**Supplementary Figure 5: The rate of TDP-43-induced SOD1 inclusion formation is decreased in the presence of 5-FUrd**. The rate of inclusion build-up per hour is based on the linear growth phase (approximately 8-24 h after data acquisition or 24-40 h post co-transfection) of TDP-43-induced SOD1 aggregation in the presence of 1 or 5 µM 5-FUrd

**Supplementary Figure 6:** **The presence of uridine does not decrease the effect of 5-FUrd to reduce SOD1 inclusion formation**. HEK293FT cells were incubated with 5-FUrd and uridine 4-6 hours after co-transfection with TDP-43^ΔNLS^ and SOD-based reporter protein. Cells were collected 48 h post-transfection, treated with saponin and analyzed using flow cytometry for presence of aggregated reporter protein. Graphs represent the percentage of total cells with inclusion compared to transfected cells treated with vehicle control (green TDP-43^ΔNLS^ column). Statistical significance was determined using one-way ANOVA and Dunnett’s test for multiple comparisons (* p <0.05; ** p <  0.01; *** p < 0.001; **** p  <  0.0001).

**Supplementary Material References**

13. E. Pokrishevsky et al., Tryptophan 32-mediated SOD1 aggregation is attenuated by pyrimidine-like compounds in living cells. Scientific Reports 8, 15590 (2018).

14. M. G. DuVal et al., Tryptophan 32 mediates SOD1 toxicity in a in vivo motor neuron model of ALS and is a promising target for small molecule therapeutics. Neurobiology of disease 124, 297-310 (2019).

22. E. Pokrishevsky, R. H. Hong, I. R. Mackenzie, N. R. Cashman, Spinal cord homogenates from SOD1 familial amyotrophic lateral sclerosis induce SOD1 aggregation in living cells. PloS one 12, e0184384-e0184384 (2017).

24. M. G. DuVal et al., Growth Differentiation Factor 6 As a Putative Risk Factor in Neuromuscular Degeneration. PloS one 9, e89183 (2014).

29. G. S. Wright et al., Purification and Structural Characterization of Aggregation-Prone Human TDP-43 Involved in Neurodegenerative Diseases. iScience 23, 101159 (2020).

34. S. Pozzi et al., Virus-mediated delivery of antibody targeting TAR DNA-binding protein-43 mitigates associated neuropathology. J Clin Invest 129, 1581-1595 (2019).

73. J. C. Stevens *et al.*, Modification of superoxide dismutase 1 (SOD1) properties by a GFP tag--implications for research into amyotrophic lateral sclerosis (ALS). *PloS one* **5**, e9541 (2010).

74. A. Wang *et al.*, A single N-terminal phosphomimic disrupts TDP-43 polymerization, phase separation, and RNA splicing. *The EMBO journal* **37** (2018).

75. K. Ginalski, A. Elofsson, D. Fischer, L. Rychlewski, 3D-Jury: a simple approach to improve protein structure predictions. *Bioinformatics (Oxford, England)* **19**, 1015-1018 (2003).

76. A. Herbert (MaxCluster: A tool for Protein Structure Comparison and Clustering.

77. S. Pronk *et al.*, GROMACS 4.5: a high-throughput and highly parallel open source molecular simulation toolkit. *Bioinformatics (Oxford, England)* **29**, 845-854 (2013).
